# A multi-omics approach to elucidate okadaic acid-induced changes in human HepaRG hepatocarcinoma cells

**DOI:** 10.1007/s00204-024-03796-1

**Published:** 2024-06-04

**Authors:** Leonie T. D. Wuerger, Heike Sprenger, Ksenia Krasikova, Markus Templin, Aaron Stahl, Uta M. Herfurth, Holger Sieg, Albert Braeuning

**Affiliations:** 1 Department of Food Safety, German Federal Institute for Risk Assessment, Berlin, Germany; 2 NMI Natural and Medical Sciences Institute at the University Tübingen, Reutlingen, Germany

**Keywords:** Okadaic acid, HepaRG cells, Liver proteome, Phosphoproteome, Multi-omics

## Abstract

Okadaic acid (OA), a prevalent marine biotoxin found in shellfish, is known for causing acute gastrointestinal symptoms. Despite its potential to reach the bloodstream and the liver, the hepatic effects of OA are not well understood, highlighting a significant research gap. This study aims to comprehensively elucidate the impact of OA on the liver by examining the transcriptome, proteome, and phosphoproteome alterations in human HepaRG liver cells exposed to non-cytotoxic OA concentrations. We employed an integrative multi-omics approach, encompassing RNA sequencing, shotgun proteomics, phosphoproteomics, and targeted DigiWest analysis. This enabled a detailed exploration of gene and protein expression changes, alongside phosphorylation patterns under OA treatment. The study reveals concentration- and time-dependent deregulation in gene and protein expression, with a significant down-regulation of xenobiotic and lipid metabolism pathways. Up-regulated pathways include actin crosslink formation and a deregulation of apoptotic pathways. Notably, our results revealed that OA, as a potent phosphatase inhibitor, induces alterations in actin filament organization. Phosphoproteomics data highlighted the importance of phosphorylation in enzyme activity regulation, particularly affecting proteins involved in the regulation of the cytoskeleton. OA's inhibition of PP2A further leads to various downstream effects, including alterations in protein translation and energy metabolism. This research expands the understanding of OA's systemic impact, emphasizing its role in modulating the phosphorylation landscape, which influences crucial cellular processes. The results underscore OA's multifaceted effects on the liver, particularly through PP2A inhibition, impacting xenobiotic metabolism, cytoskeletal dynamics, and energy homeostasis. These insights enhance our comprehension of OA's biological significance and potential health risks.

**Supplementary Information:**

The online version contains supplementary material available at 10.1007/s00204-024-03796-1.

## Introduction

Harmful algae blooms are an event of an explosive growth of phytoplanktonic algae, thereby discoloring the water. These algae produce so-called marine biotoxins. Due to climate change and human industrial waste, these harmful algae blooms occur more often in the recent years (Van Dolah 2000). The marine biotoxins can enter the human food chain by accumulating in filter-feeding shellfish, thereby posing a threat to human health. The most prevalent marine biotoxin in European waters is okadaic acid (OA), which is produced by dinoflagellates of the genus *Dinophysis* and *Prorocentrum* (EFSA 2008). They mainly produce the toxin to gain a competitive advantage against zooplankton (Gong et al. 2021).

OA is the main causative agent for diarrheic shellfish poisoning (DSP), which leads to severe gastrointestinal symptoms like stomach pain, vomiting, or diarrhea. OA is very lipophilic, thereby able to accumulate in the fatty tissue of filter-feeding shellfish (EFSA 2008). Its structure was first published by Tachibana et al*.* in 1981, after it had been isolated from the marine-sponge *Halichondria okadai* (Tachibana et al. 1981). In 1988, the inhibitory potential of OA on protein phosphatases, mainly protein phosphatases 1 and 2 (PP1 and PP2A), was published (Bialojan and Takai 1988). OA was shown to be cytotoxic (Fessard et al. 1996; Le Hégarat et al. 2006; Ferron et al. 2014) in vitro. Furthermore, it potently exerts embryotoxicity in vitro (Ehlers et al. 2010; Ariu et al. 2012), and although there has not been an evaluation in humans so far, it was able to pass the placental barrier in mice (Matias and Creppy 1996a, b). OA also shows tumor promoting properties (Suganuma et al. 1988; Suganuma et al. 1992; Fujiki and Suganuma 1993; Messner et al. 2006, Jiménez-Cárcamo et al. 2020) and has also been associated with several cases of colorectal cancer in epidemiological studies (Cordier et al. 2000; Lopez-Rodas et al. 2006; Manerio et al. 2008). Because of the clear consumer risks, the European union implemented a limit of 160 µg OA equivalents per kg shellfish (EFSA 2008). However, this was based on the acute toxic effects and did not take into account long-term effects of lower doses, especially in organs other than the intestine.

OA is actively transported from the intestine into the bloodstream (Ehlers et al. 2011). It is furthermore able to disrupt tight junction proteins, which may further contribute to an uptake of OA into the bloodstream (Dietrich et al. 2019; Huang et al. 2023). There is also evidence that OA is distributed throughout the body, with a particularly long retention time in the liver (Matias et al. 1999; Ito et al. 2002; Louzao et al. 2021). OA can also enter the enterohepatic circulation (Matias and Creppy 1996a, b). With all this evidence, it is imperative to study the effects of OA on the liver.

It was recently shown that OA is able to strongly down-regulate cytochrome P450 (CYP) enzymes at the RNA and protein levels in HepaRG cells. Furthermore, several other proteins of xenobiotic metabolism were also affected (Wuerger et al. 2022). The effect on the CYP enzymes seems to be dependent on an activation of nuclear factor kappa-light-chain-enhancer of activated B cells (NF-κB) and a subsequent activation of the Janus kinase (JAK)-signal transducer and activator of transcription (STAT) (JAK/STAT) signaling pathway through the release of several proinflammatory cytokines (Wuerger et al. 2023). However, as OA is a potent phosphatase inhibitor, we expect additional, more global effects of OA on signaling in liver. In a recent proteomics study, we were able to find effects of OA on energy homeostasis, inflammation, and signal transduction in HepaRG cells (Wuerger et al. 2023). However, this study was limited with respect to its scope and methodology. In this study, we used differentiated HepaRG cells and incubated them with non-toxic OA concentrations for different time periods. The human hepatocarcinoma cell line HepaRG can be differentiated into hepatocyte-like cells using DMSO. Differentiated HepaRG cells express liver-specific enzymes at similar levels to primary human hepatocytes (Kanebratt and Andersson 2008, Tascher et al. 2019). Therefore, they are a fitting in vitro model for our study.

Using these cells, we here provide a more advanced and comprehensive characterization of the liver proteome after OA, exposure using an integrative multi-omics approach, encompassing RNA sequencing, shotgun proteomics, phosphoproteomics, and targeted DigiWest analysis.

## Experimental procedures

### Chemicals

OA (purity ≥ 98%) was purchased from Enzo Life Sciences GmbH (Loerrach, Germany). All other standard chemicals and materials were either purchased from Sigma Aldrich (Taufkirchen, Germany) or Roth (Karlsruhe, Germany) in the highest available purity.

### Cell cultivation

HepaRG cells were purchased from Biopredic International (Saint-Grégoire, France). They were seeded in 6-well plates (2 × 10^5^ cells/well) and incubated in William’s E medium supplemented with 10% fetal bovine serum (FBS), 5 μg/mL insulin (medium and both supplements from PAN-Biotech GmbH, Aidenbach, Germany), 50 μM hydrocortisone hemisuccinate (Sigma Aldrich, Taufkirchen, Germany), 100 U/mL penicillin, and 100 μg/mL streptomycin (Capricorn Scientific, Ebsdorfergrund, Germany) for 14 days at 37 °C. Afterwards, 1% DMSO was added to the medium for 2 days to start differentiation. Then, DMSO content was increased to 1.7% for another 12 days, after which the cells were further cultivated in serum-free medium [SFM; William’s E medium without phenol red (PAN-Biotech GmbH, Aidenbach, Germany), supplemented with 100 U/mL penicillin and 100 μg/mL streptomycin, 2.5 μM hydrocortisone hemisuccinate, 10 ng/mL human hepatocyte growth factor (Biomol GmbH, Hamburg, Germany), 2 ng/mL mouse epidermal growth factor (Sigma Aldrich, Taufkirchen, Germany), and 0.5% DMSO], as adapted from Klein et al. (Klein et al. 2014) for two more days. Afterwards, the cells were incubated with 33 or 100 nM OA for up to 24 h. OA concentrations were chosen as previously described (Wuerger et al. 2022; Wuerger et al. 2023). Cells were then harvested in ice-cold PBS and centrifuged (5 min, 2000 × g, 4 °C), and the cell pellets were frozen at -80 °C until use.

### RNA preparation and sequencing

For transcriptomics analysis, the cells were washed with ice-cold PBS. Afterwards, they were lysed using ice-cold RLT buffer (RNeasy Mini Kit, Qiagen GmbH, Hilden, Germany) containing 1% β-mercaptoethanol (Merck Schuchardt OHG, Hohenbrunn, Germany). The RNA was then extracted according to the instructions of the Rneasy Mini Kit (Qiagen GmbH, Hilden, Germany). Concentration and integrity of RNA were evaluated according to the manufacturer’s protocol using the Agilent RNA 6000 Nano LabChip kit in the Agilent 2100 Bioanalyzer (Agilent, Santa Clara, USA). All RNA integrity numbers (RIN) were above 9.3. Next-generation sequencing was then carried out by Eurofins (Eurofins Scientific, Luxembourg, Luxembourg) using the Illumina NovaSeq 6000 platform, 2 × 150 bp. Demultiplexing of sequencing reads was accomplished using Illumina bcl2fastq (version 2.20). Depths of ~ 66 million paired-end 150 bp reads were generated for each sample (see Supplemental Table 1 for details). The raw RNA sequencing data are available from GEO under accession number GSE252563. Reads were classified as ribosomal RNA (rRNA) and removed by RiboDetector version 0.2.7 (Deng et al. 2022). Quality and adapter trimming was done by fastp version 0.20.0 (Chen et al. 2018). Reads were aligned to the human genome (hg38, UCSC) using STAR version 2.7.8a (Dobin et al. 2013) and quantified per gene ID by RSEM version 1.3.3 (Li and Dewey 2011).

### Shotgun LC–MS

For proteome analysis, proteins were prepared using the iST sample preparation kit (PreOmics, Planegg/Martinsried, Germany) according to the instruction manual, with one change: after lysis, the protein concentration was measured using the Bio-Rad protein assay according to the instruction manual (Bio-Rad Laboratories GmbH, Feldkirchen, Germany) against a bovine serum albumin standard curve. Based on the protein concentration, only 100 µg of protein was used for sample preparation. The resulting peptide solution was diluted 1:20 with 5% (v/v) acetonitrile containing 0.1% (v/v) formic acid. For phosphoproteome analysis, cells harvested from three independent replicates were pooled prior to iST preparation and further purified using the High-Select™ TiO_2_ Phosphopeptide Enrichment Kit (Thermo Fisher Scientific, Bremen, Germany) according to the manufacturer’s instructions. Resulting peptides were reconstituted in 100 µl 5% acetonitrile containing 0.1% formic acid.

LC–MS analysis was performed using 3 µl of the peptides or 20 µl of the phosphopeptides on an UltiMate 3000 RLSCnano, that was coupled on-line to a Q Exactive Plus mass spectrometer via Nanospray Flex Ion Source, which was operated using Xcalibur 4.4 (Thermo Fisher Scientific, Bremen, Germany). The peptides were trapped using an Acclaim PepMap 100 C18 nano viper column (0.75*20 mm, Thermo Fisher Scientific, Bremen, Germany) with a flow of 5 µl/min with 3% aq. acetonitrile containing 0.05% (v/v) trifluoroacetic acid (45 °C, 5 min) and then separated using linear gradients (starting with 0.1% (v/v) formic acid in water, the content of 80% aq. acetonitrile containing 0.1% (v/v) formic acid was increased from 5 to 35% in 90 min and then from 35 to 50% in 5 min) on an Acclaim PepMap 100 C18 nano viper column (0.75*500 mm, Thermo Fisher Scientific, Bremen, Germany; 0.3 µl/min, 45 °C). After separation, the eluates were evaporated and ionized using a stainless-steel emitter (Thermo Fisher Scientific, Bremen, Germany). Analysis was performed using data-dependent acquisition (DDA) mode. To maximize identification outcome, samples were injected once and a brief database search using SEQUEST HT was performed. Based on these results, a time-dependent (± 1 min) exclusion list, which was extended to z = 2, 3, 4, was generated. Afterwards, each sample was analyzed in triplicates.

Raw data were analyzed using the ProteomeDiscoverer software (Thermo Fisher Scientific, Bremen, Germany; version 2.4.1.15). Human reference protein accessed at UniProt (UP000005640; date 17/09/2021) was used for identification using SEQUEST HT as search engine with a maximum missed cleavage of 2, and a precursor mass and a fragment mass tolerance of 10 ppm. B- and y-ions were both weighted at 1. Oxidation, carbamidomethyl, acetylation, and phosphorylation were selected as dynamic modifications. Precursor ions were quantified using the Precursor Ion quantifier node based on the area under the peak. The validation was based on the q-value. False discovery rates (FDR) for peptide and protein identification were set to 1% strict and 5% relaxed. For phosphopeptide identification, the PTM RS node was additionally used. Only proteins identified with at least two peptides were used for further analysis. The mass spectrometry proteomics data have been deposited to the ProteomeXchange Consortium via the PRIDE (Perez-Riverol et al. 2022) partner repository with the dataset identifier PXD048968.

### DigiWest®

HepaRG cells were seeded into 25 cm^2^ flasks and treated as described above. Afterwards, cells were pelleted and frozen in liquid nitrogen until further use. DigiWest® was performed as described before (Treindl et al. 2016). Three independent replicates were pooled prior to the measurements. The original dataset consisting of 182 entries was filtered by removing redundant entries and those below the limit of quantification resulting in 132 unique analytes.

### Experimental design and statistical rationale

In this study, we aimed to integrate RNA sequencing, shotgun proteomics, phosphoproteomics, and a targeted DigiWest to gain a comprehensive picture of OA-induced changes in human HepaRG cells. To account for variability, each analysis initially used three biological replicates. Due to sample constraints, however, the biological replicates were pooled prior to the phosphoproteome and DigiWest analyses. This pooling was necessary to meet the sensitivity and depth required for these methods. Despite this limitation, we attempted to mitigate the impact by ensuring comprehensive coverage and reproducibility in our RNA sequencing and proteomic analyses using individual replicates. Samples were collected after 0.5, 4, 12, and 24 h. To account for technical variability, three technical replicates were created in the shotgun and phosphoproteome analyses. The detailed statistical rationale for each analysis is described below in the section “Bioinformatic analysis and statistics”.

### Bioinformatic analysis and statistics

After removing genes with low expression (sum of reads across all samples below ten), the retained 22,122 genes were analyzed in R version 4.3.2 (R Core Team 2020) using package DESeq2 version 1.42.0 (Love et al. 2014) using default settings for estimation of size factors and dispersion. Negative Binomial GLM fitting and Wald statistics were applied to test for differential gene expression between each treatment and control conditions, respectively. False discovery rate (FDR) was used to control for multiple testing (Benjamini and Hochberg 1995). Only genes with an adjusted p value < 0.1 and |log_2_FC|> 1 were identified as DEGs and included in the further analyses. Variance stabilizing transformation was applied prior to probabilistic PCA (ppca) on centered data by the R-package pcaMethods version 1.94.0 (Stacklies et al. 2007).

Shotgun proteomics and phosphoproteomics data were filtered for common contaminants like human keratin and proteins with very high NA count were removed, so that 3028 unique proteins and 674 unique peptides were retained, respectively. Phosphoproteomics data were filtered for peptides with a phosphorylation as modification (288 unique entries) and log_2_FC was calculated relative to the negative control per timepoint. To calculate the actual change of phosphorylation upon OA treatment (in contrast to changed abundances), phosphopeptides were matched to peptides in the Shotgun proteomics dataset and the ratio of both log_2_FC values was calculated. Statistical significance of Shotgun proteomic changes was determined by Student’s *t* tests and FDR for multiple testing control (cutoffs: adjusted *p* value < 0.05 and |log_2_FC|> 0.5). However, the DigiWest and phosphoproteomic data included only one sample per condition (three biological replicates pooled). Thus, statistical significance could not be estimated and |log_2_FC|> 1 was set as cutoff to define relevant changes.

OmniPathR R-package version 3.10.1 (Türei et al. 2016) was used to collect protein–protein and kinase interactions for selected proteins of interest (POI) and phosphatases, followed by building a network with the shortest path via intermediate regulators using the R-package igraph version 1.6.0 (Csardi and Nepusz 2005). POI for actin cytoskeleton organization were selected based on the Gene Ontology (GO) terms “actin filament binding” (GO:0051015), “actin filament organization” (GO:0007015), “actin cytoskeleton” (GO:0015629), and “actin cytoskeleton organization” (GO:0030036).

Kinase-substrate enrichment analysis (KSEA) was performed using the KSEA App (https://casecpb.shinyapps.io/ksea/) using PhosphoSitePlus and NetworKIN (NetworKIN score cutoff = 1) as the kinase-substrate dataset. Log_2_FC ratios of phosphopeptides served as input and Z-Scores were used as output for further visualization (Casado 2013; Wiredja et al. 2017). Cytoscape version 3.10.1 was used to create a signaling network including selected protein kinases based on the apps OmniPath version 2.3, PathLinker version 1.4.3 and Omics Visualizer version 1.3.1 where Z-Scores from KSEA and log_2_FC values are overlayed to nodes.

GO term enrichment analysis was performed using R-package clusterProfiler version 4.10.0 (Wu et al. 2021), specifically the “compareCluster” function to compare multiple conditions and visualized by dot-plots of the top enriched terms. Heatmaps were created with the R-package ComplexHeatmap version 2.18.0 (Gu et al. 2016) using default settings if not mentioned otherwise. K-means clustering was performed by the R-package stats for various omics data as input with manually chosen number of centers. For pathway analysis, transcriptomic data were analyzed through the use of Ingenuity Pathway Analysis (IPA; QIAGEN Inc., https://digitalinsights.qiagen.com/products-overview/discovery-insights-portfolio/analysis-and-visualization/qiagen-ipa/; IPA Winter Release December 2023).

## Results

### Transcriptomics

**Fig. 1:**
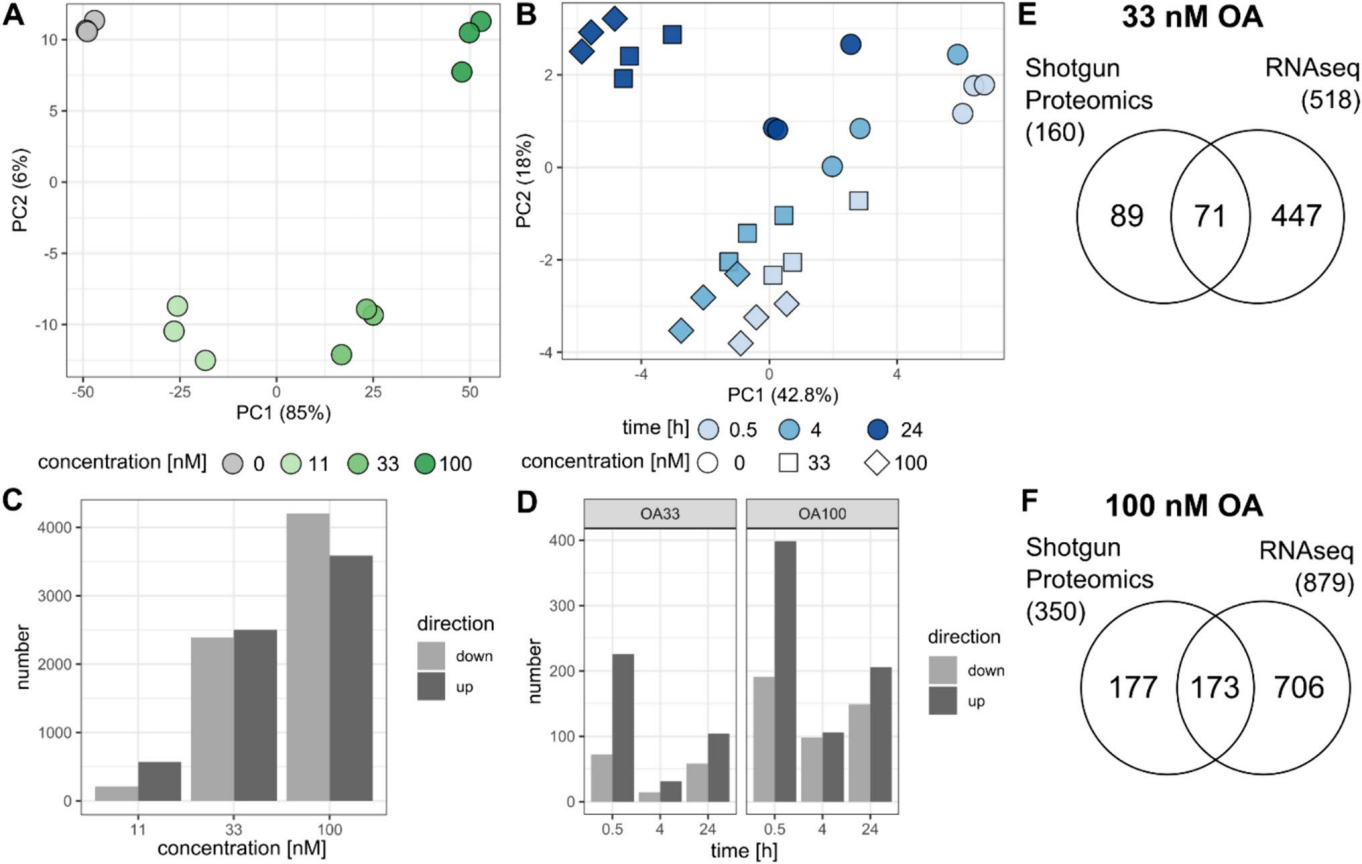
Global omics data results for transcriptomics and proteomics. Scores plots of principal component analysis (PCA) for transcriptomics (**A**) and shotgun proteomics (**B**). Colors indicate the different treatments: negative control = 0 nM OA (gray), 11 nM OA (light green), 33 nM OA (green), 100 nM OA (dark green) for transcriptomics; incubation time of 0.5 h (light blue), 4 h (blue), 24 h (dark blue) and negative control (circle), 33 nM OA (square), 100 nM OA (diamonds) for proteomics. Bar plots for number of significantly changed genes (**C**) and proteins (**D**). Genes with p_adj_ < 0.1 and |log_2_FC|> 1 were considered as significant and proteins with p_adj_ < 0.05 and |log_2_FC|> 0.5. Venn diagrams comparing significantly regulated genes/proteins for treatment with 33 nM (**E**) and 100 nM OA (**F**)

To detect gene expression changes upon okadaic acid (OA) treatment for 24 h in HepaRG cells, we performed RNA sequencing and differential gene expression (DGE) analysis. As described in the Materials and methods section, raw counts were filtered resulting in 22,122 genes expressed above the threshold. After normalization and transformation of processed counts data, Principal Component Analysis (PCA) was used to visualize and reduce the complexity of the dataset. Figure 1A shows the PCA scores plot for all twelve samples analyzed by RNAseq. PC1 and PC2 representing most of the variance clearly separate the samples treated with an increasing concentration of OA from the negative control. This is also reflected by the number of differentially expressed genes (DEGs) that peaks at 4200 down- and 3586 up-regulated upon treatment with 100 nM OA (Fig. 1C). We found that 184 and 513 genes were significantly down- and up-regulated in common between all concentrations of OA treatment, respectively (Supplemental Fig. 1). Overall, there were 8181 genes that were significantly changed in their expression in at least one condition which reflects 36.9% of the studied genes (Supplemental Table S5).

**Fig. 2:**
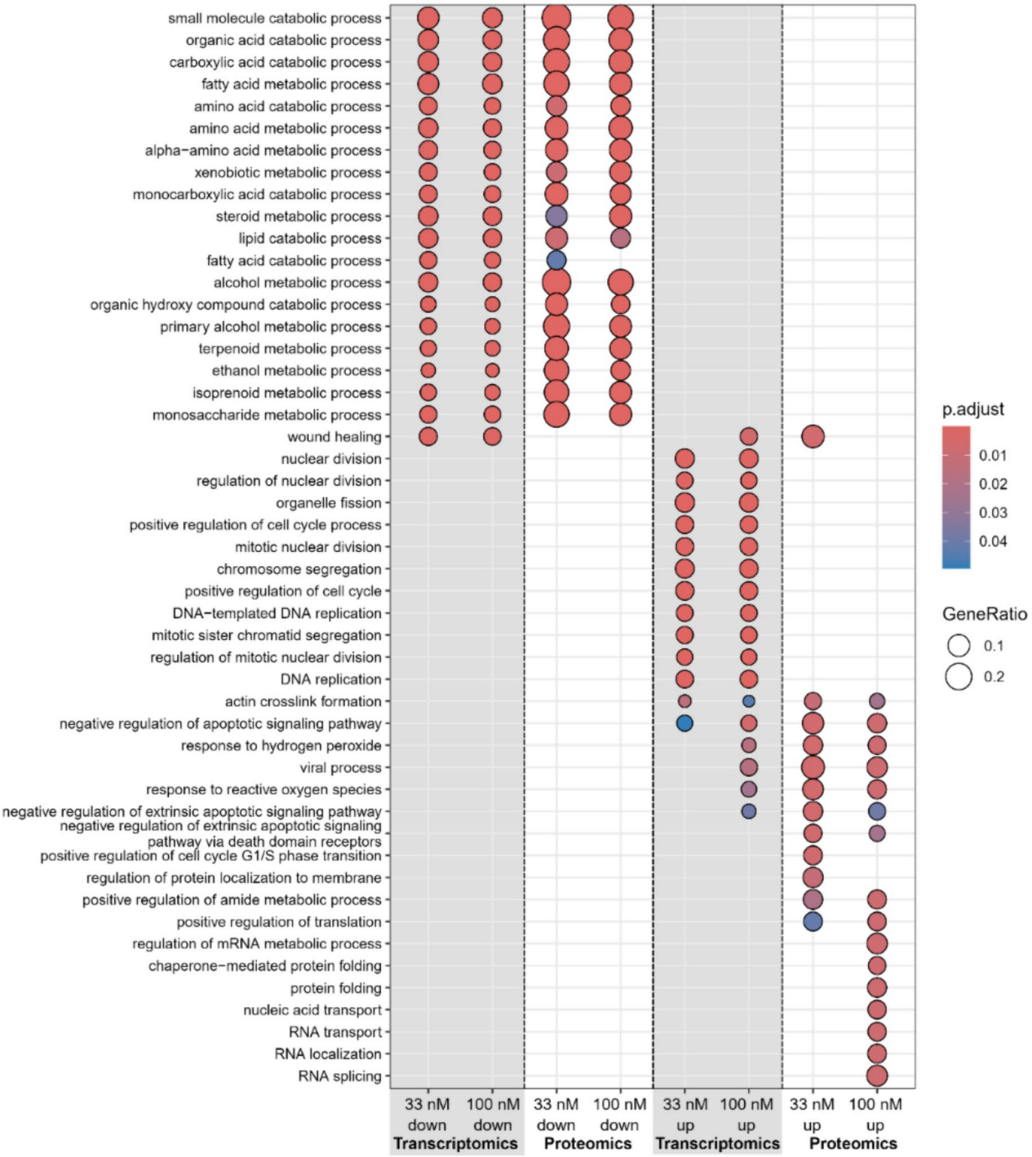
Dot-plot of enriched GO terms for transcriptomics (RNAseq) and Shotgun proteomics. Only results for OA treatment (33, 100 nM) after 24 h are depicted and split by up-/down-regulated genes/proteins. The color indicates the significance measured by the adjusted BH p value and the dot size indicates the gene ratio per GO term

**Fig. 3:**
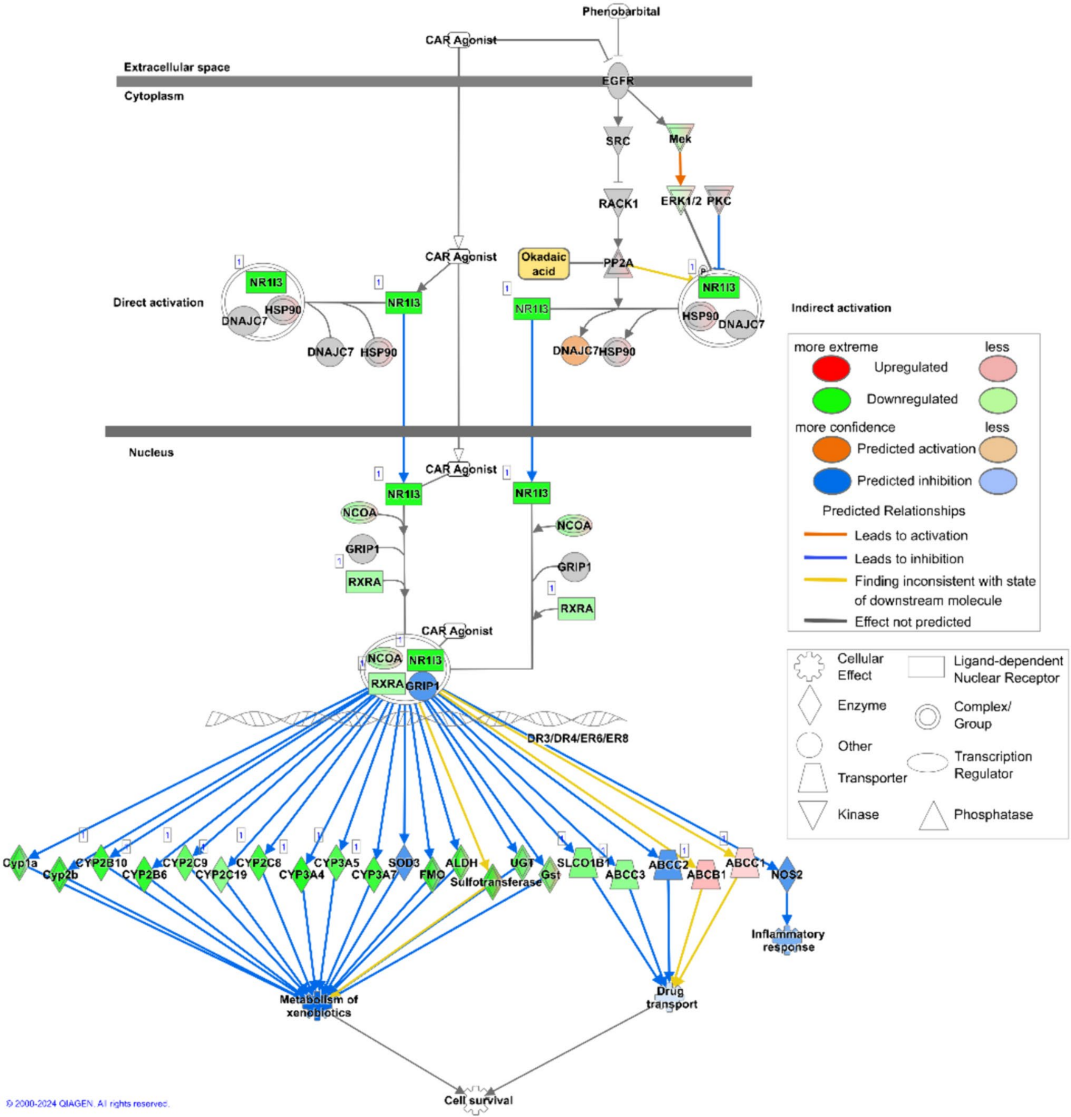
Overview of xenobiotic metabolism regulated by the CAR signaling pathway from Ingenuity Pathway Analysis (IPA). Transcriptome data (log_2_FC values) for treatment with 33 nM OA (24 h) were mapped while results for 100 nM OA (24 h) showed a similar picture (not shown). CAR is labeled by its synonym NR1I3. Genes detected in our dataset are shown in green (down-regulated) or red (up-regulated). Genes shown in orange or blue represent a predicted up- (orange) or down-regulation (blue)

In our study, GO term enrichment analysis was performed to identify significant biological processes affected by different concentrations of OA. In summary of the dot-plot in Fig. 2, the analysis highlighted that genes up-regulated at 33 nM were predominantly associated with cell cycle regulation and DNA replication. At higher concentrations (100 nM) also GO terms related to reactive oxygen species (ROS) and apoptosis signaling pathways were associated (Fig. 2). Down-regulated genes were consistently enriched for fatty acid and xenobiotic metabolism for both OA concentrations. The underlying CAR signaling pathway is depicted for OA 33 nM treatment based on top “canonical pathways” results from Ingenuity Pathway Analysis (IPA) in Fig. 3 with key regulators, such as RXRA, NCOA, and CAR (NR1I3) itself showing decreased gene expression. CAR-regulated targets, including CYPs, UGTs, and transporters (e.g., SLCO1B1, ABCC3), are also down-regulated leading to an inactivation of the downstream xenobiotic metabolism.

### Shotgun proteomics

In addition to RNA sequencing, we also carried out Shotgun Proteomics analysis to identify proteins that were affected by OA (33 and 100 nM) in their expression. As the transcriptomic analysis focused exclusively on the changes in signaling pathways after 24 h, we wanted to include the relevant protein changes at earlier time points (0.5 and 4 h) as well. After processing and filtering the dataset as described in the Materials and methods section, 3028 unique proteins were detected and quantified. The PCA scores plot for all 27 samples analyzed by Shotgun Proteomics, depicted in Fig. 1B, clusters the samples along PC1 and PC2 based on factors of concentration and time. The samples of each time point are separated by the increasing OA concentration, as the samples of 100 nM are more distant from the negative control than those of 33 nM. The treatment effects on the proteome for 0.5 and 4 h seem to be more similar to each other as the samples are clustering together, while those for 24 h treatment were separated.

Subsequently, each OA treatment was compared to the negative control of the respective time point using Student’s *t* test, and significant protein changes were considered for adjusted *p* values < 0.05 and |log_2_FC|> 0.5. In general, the percentage of differentially expressed proteins (DEPs) was 31.3% (947 proteins changed in at least one condition), which is quite similar to the mRNA level even if the absolute numbers are lower.

In agreement with the PCA scores plot, the concentration-dependent effect on the number of DEPs is also indicated in Fig. 1D as already observed for DEGs (Fig. 1C). When comparing the DEGs identified by RNAseq with deregulated proteins, we found an overlap of 71 and 173 molecules for treatment with 33 and 100 nM OA, respectively (Fig. 1E–F). For instance, SERPINB2, ITGA2, and SLC1A5 were commonly up-regulated, while CYP2E1, FABP1, and ADH1B were down-regulated at OA both levels. Interestingly, the extent of up- or down-regulation seemed to be higher at the transcriptomic than at the proteomic level which is apparent from the heatmaps in Supplemental Fig. 2. Indeed, correlation analysis of log_2_FC values (100 nM OA) of both omics levels results in a correlation coefficient of 0.79 and a slope of 2.38 for linear model of transcriptomics in dependence of proteomics. Also, for 33 nM OA, a factor > 2 was derived for this significant relationship, as summarized in Supplemental Fig. 3. Besides this set of commonly regulated molecules, each level showed specifically deregulated genes or proteins that were not identified by the other method.

Next, we analyzed the overlaps between sets of DEPs and visualized them in Venn diagrams. For both OA concentrations, they indicate rather distinct proteomic changes at the early stage (0.5 and 4 h) and late stage (24 h) as there are many specific proteins at each condition and few common DEPs (Supplemental Fig. 4).

**Fig. 4:**
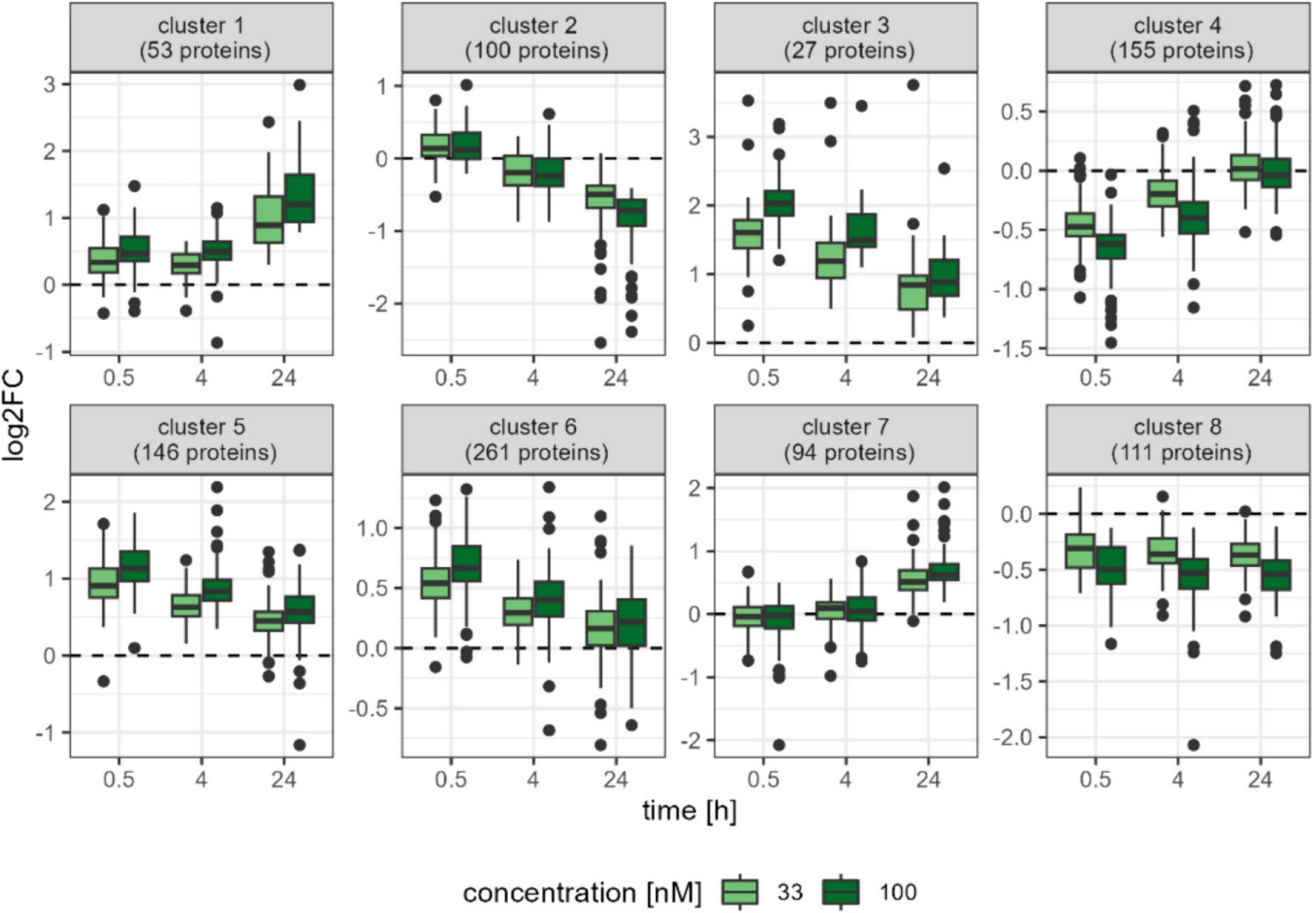
Boxplot of log_2_FC across eight clusters of DEPs measured by shotgun proteomics over time and per OA concentration (33 nM in green, 100 nM in dark green) according to kmeans clustering

To get a better understanding of the biological pathways affected by OA treatment over time, we performed kmeans clustering analysis to group all 947 DEPs into eight clusters. The boxplots in Fig. 4 show the different concentration- and time-dependent proteomic changes and the enriched GO terms associated with each cluster are summarized in Supplemental Table 2. Cluster 2 contains 100 proteins, such as CYP2E1, ABCC3, and FABP1, that are mainly down-regulated after 24 h and enriched for GO terms related to xenobiotic and lipid metabolism (Supplemental Fig. 5A). However, cluster 4 reflects an early proteomic response including 155 down-regulated proteins that are associated with ribosome biogenesis and translation, e.g., RRP8, RPL28, and EIF5B. Multiple clusters indicate different patterns of up-regulated proteins, as clusters 1 and 7 are reaching the maximum expression after 24 h, while clusters 3, 5, and 6 are showing early proteomic changes upon OA treatment. Clusters 3, 5, and 6 are mainly enriched for biological processes like RNA splicing and fatty acid metabolism; on the other hand, proteins in cluster 1 and 7 are associated with apoptosis and mRNA stabilization, respectively. Moreover, proteins involved in actin filament organization appear in cluster 6 (early up-regulation, e.g., CTNNA2, PAK1, and SPTBN4) and 7 (late up-regulation, e.g., MARCKS, STMN1, and ZYX), as summarized in Supplemental Fig. 5B.

**Fig. 5:**
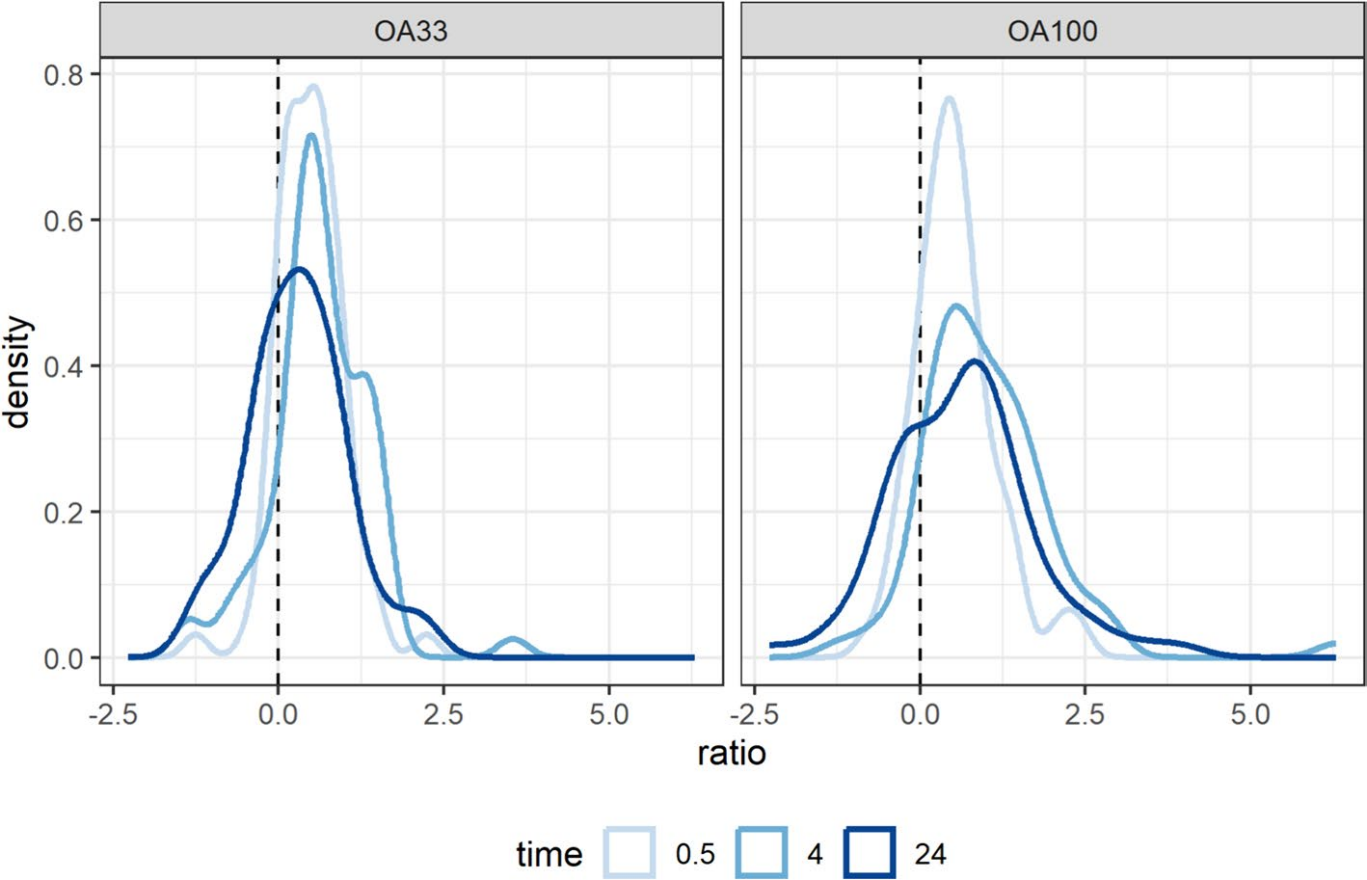
Distribution of log_2_FC ratios for OA treatment for 0.5, 4 and 24 h in the phosphoproteome dataset. The left side shows the density plot for 33 nM OA (OA33) and the right side for 100 nM OA (OA100)

### Phosphoproteomics

As a potent phosphatase inhibitor of mainly PP1 and PP2A, effects of OA on the phosphorylation status of the cells were expected. To investigate the signaling events associated with OA treatment and subsequent PP1 and PP2A inhibition, we performed LC–MS-based profiling of enriched phosphopeptides. In total, we detected 288 unique phosphopeptides that were used as input for a PCA plot that shows a clear separation of samples according to concentration and duration of the OA treatment (Supplemental Fig. 6A). To distinguish changed phosphoprotein abundances from altered phosphorylation status, we performed a pairwise normalization of enriched phosphopeptides relative to their counterparts in the non-enriched shotgun proteomics dataset. From the total number of 288 phosphopeptides, 72 could be matched to peptides from the shotgun data which resulted in log_2_FC ratios.

**Fig. 6:**
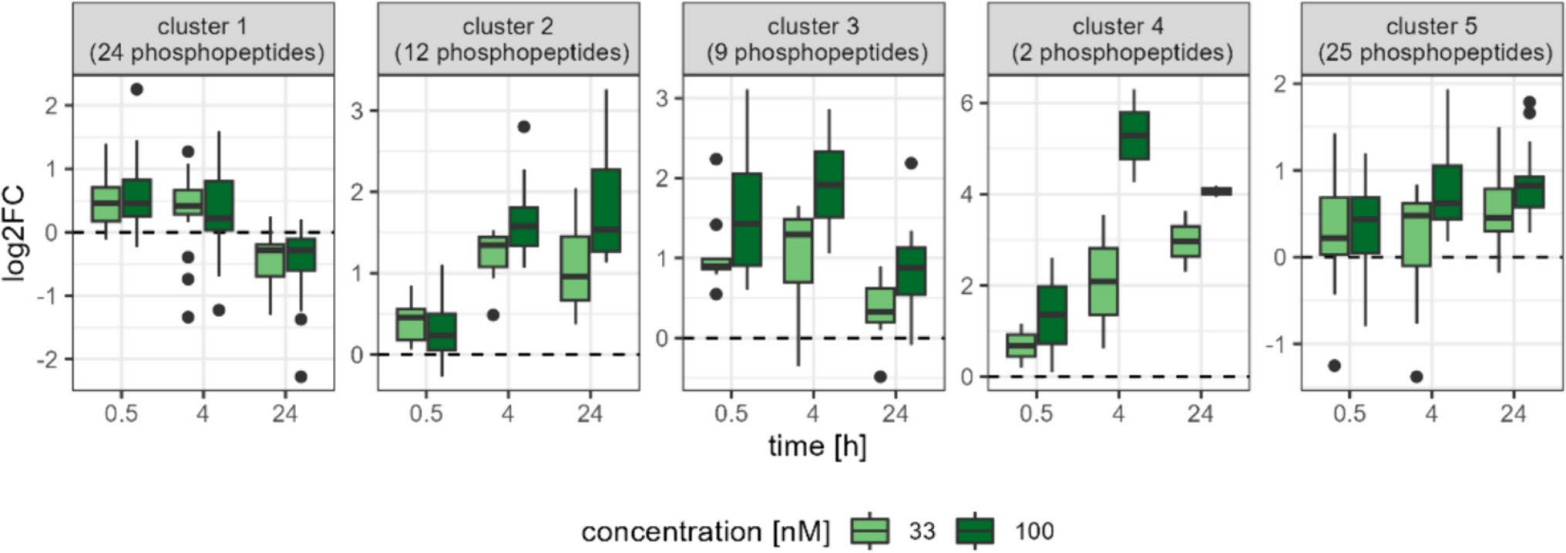
Boxplot of log_2_FC ratio across five clusters of phosphopeptides over time and per OA concentration (33 nM in green, 100 nM in dark green) according to kmeans clustering

Strikingly, OA treatment results in a global increase in phosphorylation status as indicated by the shift of log_2_FC ratios to the positive values in Fig. 5. This effect is even more pronounced for treatment with 100 nM OA compared to 30 nM with median values of 0.631 and 0.490, respectively (see Supplemental Table 3 for details). This observation is also reflected by a higher number of phosphopeptides with increased log_2_FC ratios than decreased values (Supplemental Fig. 7A) peaking after 4 h of OA treatment.

**Fig. 7:**
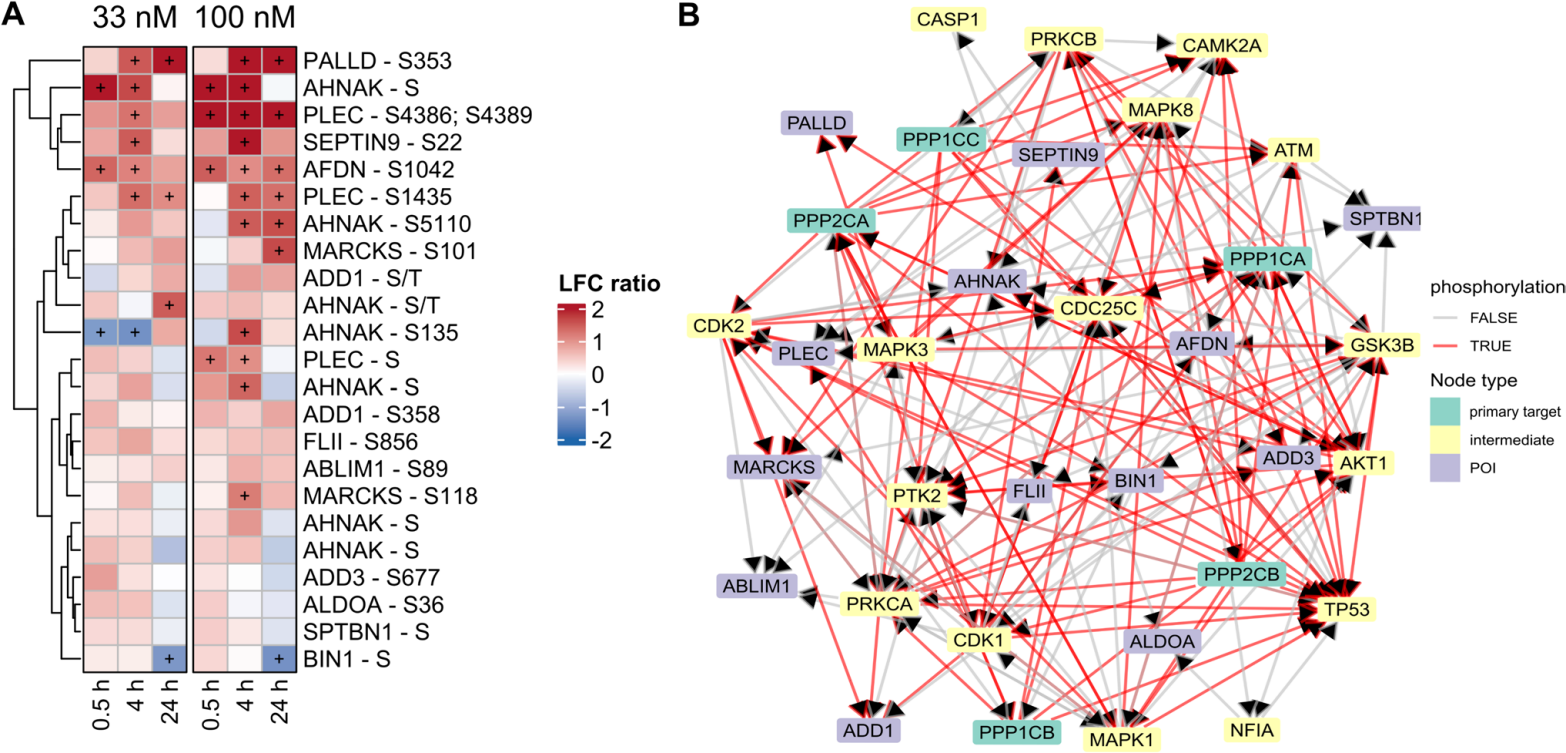
Phosphoproteomic changes connected to actin cytoskeleton. Heatmap of selected phosphopeptides related to actin filament organization (**A**). Relevant changes with |log_2_FC ratio|> 1 are indicated by “ + ” sign. Prior knowledge-based signaling network based on protein–protein interactions (PPIs) and kinase information from OmniPath for selected proteins (**B**). These proteins of interest (POI) were selected based on GO term annotation related to actin filament binding and organization. Additionally, phosphatases PP1 and PP2A (“primary targets”) as well as intermediate kinases are included in the network, and red edges indicate (de)phosphorylations in contrast to PPIs

To get a better understanding of the patterns in the phosphoproteome data, we analyzed the 72 matched phosphopeptides by kmeans clustering (Fig. 6). The five resulting groups reflect similarly reacting phosphopeptides upon OA treatment, such as cluster 1 containing 24 molecules (e.g., ADD3, PLEC, and SPTBN1) that accumulated phosphorylations after 0.5 h and 4 h, but this effect reversed after 24 h. Cluster 2 is also showing an increased phosphorylation status but not immediately, rather after 4 or 24 h with proteins like MTDH, NDRG2, and EIF4B. The biggest increase was observed for FKBP5 and USP5 in cluster 4. Across the entire dataset, there is a striking overrepresentation of proteins associated to actin filament organization, microtubule, and cadherin binding, highlighting a broad impact of OA treatment beyond the specific clusters identified.

Based on GO term annotation related to actin cytoskeleton, a subset of genes was selected for a more detailed inspection. Especially, after 4 h and 24 h, OA treatments lead to a pronounced increase of phosphorylation for PALLD, PLEC, AFDN, MARCKS, AHNAK, and SEPTIN9 (Fig. 7A). To get a better understanding of the signaling network around the proteins of interest existing knowledge for protein–protein interactions (PPIs) and kinase–substrate relationships from OmniPath utilized. The derived network in Fig. 7B shows the interactions between the primary OA targets PP1 and PP2A, selected proteins of interest (POI) and kinases as intermediate regulators. Among the most connected kinases in the network, we identified MAPK1 (ERK2), MAPK3 (ERK1), AKT1, CDK1, CDK2, GSK3B, TP53, PRKCA, and PRKCB as potential key regulators for POI that are associated with downstream effects in the actin cytoskeleton organization. Then, we explored phosphopeptides related to microtubule and cadherin binding that partially overlapped with POI for actin binding. Additionally, we recognized NDRG1 and DYNC1I2 as phosphorylated POI for microtubule organization and CTNND1 and PPL for cadherin binding/focal adhesion (Supplemental Fig. 8).

**Fig. 8:**
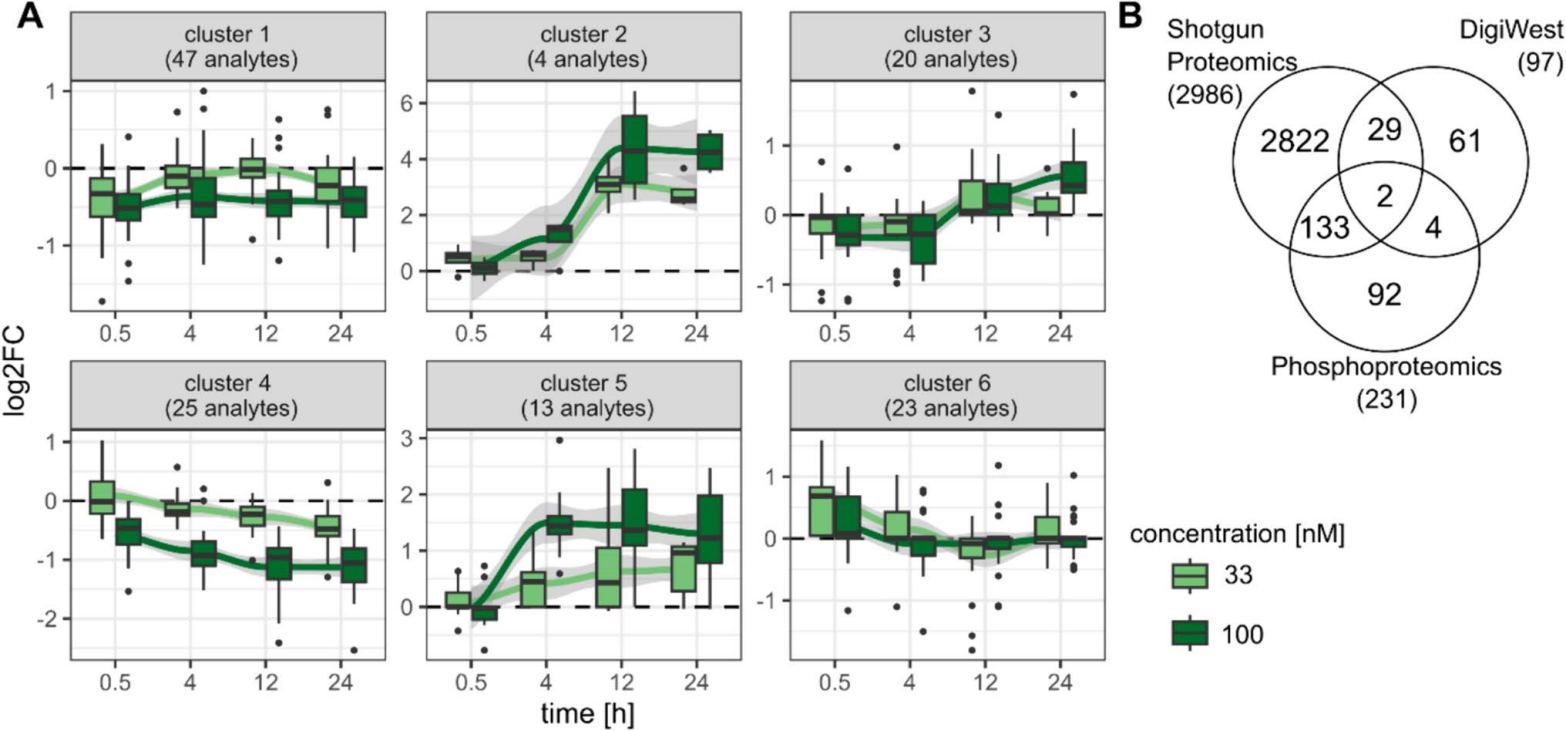
Overview of DigiWest results. Boxplot of ratio across six clusters of 136 analytes measured by DigiWest over time and per OA concentration (**A**). Venn diagram comparing the unique proteins detected by Shotgun Proteomics, Phosphoproteomics, and DigiWest (**B**)

### DigiWest (targeted proteins)

In a targeted approach, we used the targeted DigiWest method to complement the previous untargeted (phospho)-proteomic methods, covering 132 analytes that correspond to 97 unique proteins (some with different modifications). Fig. 8B illustrates that the majority of those proteins (63%) could exclusively be detected by DigiWest. Similar to the phosphoproteome data, samples cluster according to OA concentration and treatment duration in the PCA scores plot (Supplemental Fig. 6B). This is also exemplified by the rising number of down- or up-regulated analytes (|log_2_FC|> 1) with increasing OA concentration and over time (Supplemental Fig. 7B). However, we observed an early regulation at the proteome level already 0.5 h after OA treatment, which is in agreement with the Shotgun Proteomics data (Fig. 1D).

Kmeans clustering was applied to group similarly behaving analytes into groups as summarized by boxplots in Fig. 8A. Cluster 2 and 5 show strong accumulation upon OA treatment of 12 and 24 h, especially for 100 nM. Among them are the phosphorylated proteins RPS6, JUN and RB (cluster 2) as well as MAPK/CDK substrates, GSK3A/B, and FOXO3 (cluster 5). In contrast, cluster 4 reflects a moderate down-regulation of 25 analytes after longer OA 100 nM treatment, such as CYP2B6, MDM2, PTEN, NFκB2, and DVL3. Early down-regulation of PAK1/2, FOXO1, and AKT1 is depicted in cluster 1 and 3, while cluster 6 shows the opposite; namely early up-regulation of PIK3R1, ERK2, HSP27, and RICTOR (Supplemental Fig. 9).

**Fig. 9:**
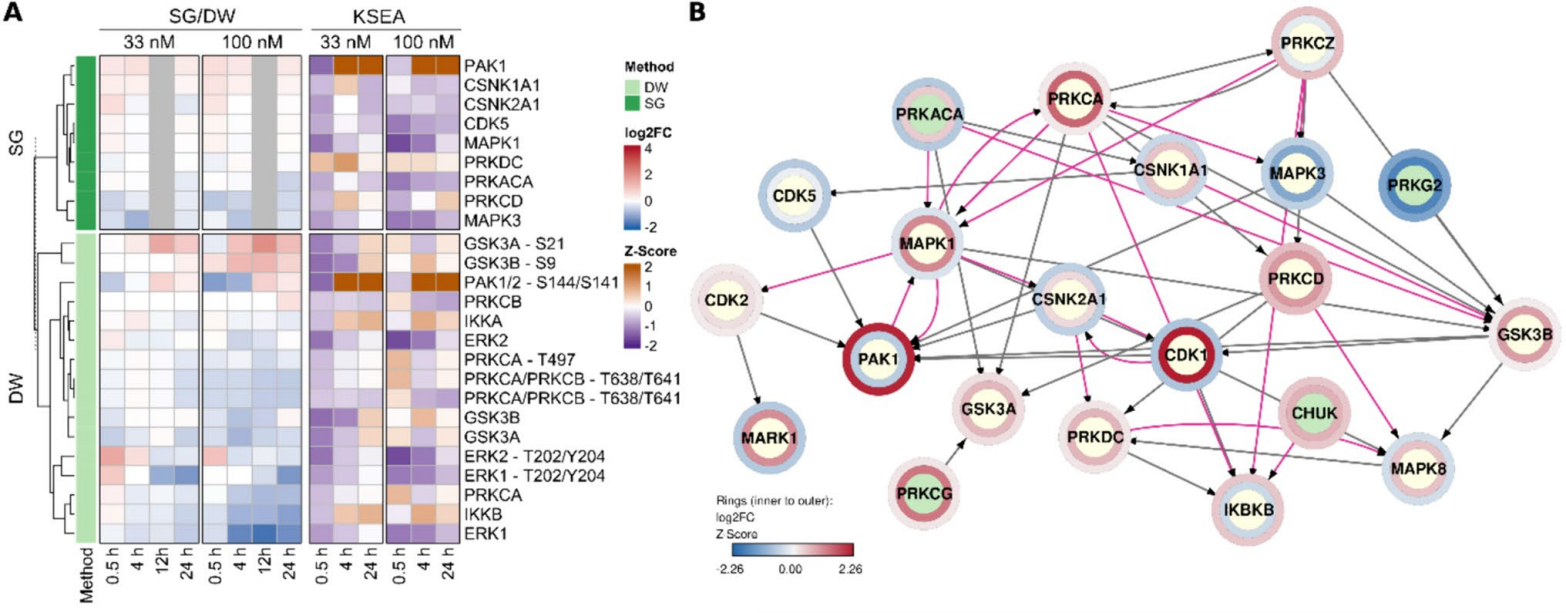
Integrative analysis of protein kinase relationships. Summary of the Kinase-Substrate Enrichment Analysis (KSEA) in combination with kinase measurements by Shotgun Proteomics and DigiWest (**A**). KSEA (right side) results in Z-Scores where positive values indicate elevated kinase activity (orange) and negative values reduced activity (purple). The left side shows log_2_FC values from DigiWest (DW) and Shotgun Proteomics for matching protein kinases. Signaling network of selected protein kinases from Cytoscape using the apps OminiPath, PathLinker and Omics Visualizer (**B**). The inner ring shows the log_2_FC values measured for OA 100 nM treatment, 24 h by RNAseq and the outer ring the Z-Score from KSEA for the matching condition and kinase. Pink edges indicate stimulation in contrast to dark grey edges for inhibition

A comparison of proteins detected by Shotgun Proteomics and DigiWest yielded an overlap of 31 analytes that was explored in more detail by a heatmap (Supplemental Fig. 10). As expected, the similarity between DigiWest and Shotgun Proteomics was higher to each other than to RNAseq log_2_FC values. The protein RPS6 was detected in the DigiWest in the form of different phosphorylated variants and showed a clear up-regulation, while the corresponding gene was slightly down-regulated. In contrast, the bottom of the heatmap depicts a group of down-regulated proteins (e.g., DEPTOR3, MAPK3/ERK1, IDH, and RAD23B), for which transcriptomic and proteomic data are in good agreement. Remarkably, this heatmap features many genes/proteins related to the NF-κB signaling pathway, such as NFKB2, EIF4E, EIF2S1, CREB1, PDK1, and PAK2, as well multiple MAP kinases. All of them are significantly up-regulated at the transcriptomic level, while the proteomic level is not giving such a clear picture. NFKB2, EIF4E (S209), and CREB1 (S133) are increased after 24 h and MAPK1/ERK2 (T202/Y204) is increased already after 0.5 h. However, the (phospho-)protein levels of the other molecules are unchanged upon treatment or in some cases decreased after 0.5 h, e.g., PAK1/2 (S144/S141) and EIF2S1 (S51).

### Integrative phosphorylation analysis

To identify which kinases might be responsible for downstream phosphorylation changes, we applied Kinase-Substrate Enrichment Analysis (KSEA). The top overrepresented kinases were PAK1, CAMK2A, AURKB, and CDK1 based on the Z-Score (Supplemental Table 4). Subsequently, Shotgun Proteomics and DigiWest data were matched to KSEA results and summarized in a heatmap (Fig. 9A) of log_2_FC values and corresponding Z-Scores. Most strikingly, PAK1 (S144/S141) accumulated over time as measured by DigiWest, which is reflected by an increasing Z-Score. In contrast, KSEA revealed decreased activity of MAPK3 (ERK1), which is in line with the down-regulation upon OA treatment according to DigiWest and Shotgun Proteomics. Furthermore, DigiWest data point to a slight decrease of GSK3A and GSK3B protein levels, while the phosphorylated forms of those kinases (GSK3A-S21, GSK3B-S9) accumulated over time, especially at 100 nM OA. KSEA hints to an early inhibition of GSK3A and GSK3B but slight activation after 24 h.

RNAseq results were integrated with Z-Scores of KSEA in a knowledge-based signaling network for OA 100 nM treatment (Fig. 9B). Overall, this network includes many kinases involved in MAPK, Wnt, or NF-κB signaling, such as MAPK1, MAPK8, PRKCA, PRKCG, CNSK1A1, GSK3A, GSK3B, IKBKB, and CHUK (= IKKA) as well as cytoskeleton organization similar to the network in Fig. 7B. MAPK3 (= ERK1) and PRKG2 were down-regulated at the transcriptomic level and KSEA also points to an inactivation, while PRKCD and CHUK (= IKKA) were up-regulated and KSEA hints to the same direction. However, there are a number of kinases, such as CK1, PAK1, and MARK1, for which gene expression and predicted kinase activity are somewhat contradictory, probably due to other regulatory mechanisms which are opposing the correlation between mRNA and protein levels.

## Discussion

The effects of OA on the liver are not very well understood so far. We recently published a proteomics study regarding the effects of OA on HepaRG cells using 2D-PAGE (Wuerger et al. 2023). In this publication, we were able to link OA exposure of HepaRG cells to proteins involved in energy metabolism, oxidative stress, protein metabolism, and the immune response. This study, however, was limited due to methodological issues.

Therefore, we now conducted a much broader, more comprehensive study covering not only the proteome, but also the transcriptome of HepaRG cells after exposure to non-cytotoxic concentrations of OA. To the best of our knowledge, this study is the first of its kind regarding the human liver cell proteome and transcriptome after OA exposure. With OA being a potent inhibitor of PP2A (Bialojan and Takai 1988), we expected OA-induced changes in the protein phosphorylation of the cells and thereby changes in signal transduction. Therefore, we expanded the study to include the phosphoproteome as well, using an LC–MS approach and a targeted DigiWest analysis regarding important signaling pathways that are regulated by phosphorylation. Based on the total number of regulated genes and proteins, there is a clear concentration dependency of the strength of deregulation in genes or proteins. Furthermore, the proteome changes are time-dependent as well. Clustering of the proteome dataset revealed a time-dependent down-regulation of lipid metabolism and an increasing up-regulation of proteins involved in actin filament organization over time.

The comparison of the GO term analysis of the transcriptome and proteome datasets revealed a strong overlap regarding the function of the down-regulated genes and proteins, with the most strongly down-regulated pathways being xenobiotic metabolism and lipid metabolism in both datasets. These data are in congruence with our previous results. In our previous proteomics study using the 2D-PAGE, we were also able to see a strong effect of OA on lipid metabolism, with the majority of the deregulated proteins involved in lipid metabolism identified being down-regulated, as well (Wuerger et al. 2023). In another 2D-study regarding the liver proteome in mice after OA exposure, Wang et al. also found the deregulated proteins assigned to the lipid metabolism to be down-regulated (Wang et al. 2021). Regarding the effect on xenobiotic metabolism, we were already able to show a strong down-regulating effect of OA on CYP enzymes, which are a very important group of enzymes in xenobiotic metabolism, and on their key regulators, the transcription factors PXR and RXRα (Wuerger et al. 2022; Wuerger et al. 2023). Functionally, this is related to the induction of inflammation by OA in liver cells. OA activates NF-κB, which then activates the inflammatory response by acting as a transcription factor for several proinflammatory cytokines (Wuerger et al. 2023). Induction of inflammation has been identified to cause changes in xenobiotic metabolism (Keller et al. 2016; Tanner et al. 2018). The induction of inflammation and a subsequent down-regulation of CYP enzymes could be recently verified in HepaRG cells by us. We further recently showed an effect on the nuclear receptors retinoid X receptor alpha (RXRα) and pregnane X receptor (PXR). However, the effect on the constitutive androstane receptor (CAR) was not studied (Wuerger et al. 2023). This study now shows an additional inhibitory property of OA on the CAR signaling pathway, which supports previous studies (Kawamoto et al. 1999; Yoshinari et al. 2003; Hosseinpour et al. 2006). Mechanistically, CAR activation depends on its phosphorylation state and is regulated by PP2A (Yoshinari et al. 2003, Shizu et al. 2017, Yokobori et al. 2019). Therefore, by inhibiting PP2A, OA might also inhibit CAR activation.

The effects of OA on the CYP enzymes and nuclear receptors was recently reviewed in detail by us. We were able to show that the majority of studies point to an inhibition of xenobiotic metabolism by OA (Wuerger et al. 2024). Evaluation of the top up-regulated pathways of the GO term analysis revealed up-regulated cell cycle regulation and, at higher concentrations, apoptosis signaling for the transcriptomics data. A similar result regarding the up-regulation of apoptotic pathways on the transcriptome level by OA was previously described by Fieber et al. They found a stimulation of apoptosis at higher OA concentrations in HepG2 cells. They further found an up-regulation of genes involved in cell cycle regulation, which is congruent with our results (Fieber et al. 2012). The upregulation of cell cycle regulation on transcriptome level by OA was also observed by Huguet et al. in Caco-2 cells (Huguet et al. 2020).

An effect of OA on the cytoskeleton has been described before (Berven et al. 2001; Huang et al. 2013; Huang et al. 2023; Dietrich et al. 2019). This study now provides deeper insight into the possible mechanisms involved in depolymerization of the polymer F-actin upon OA exposure. As seen in clusters 6 and 7 of the proteome analysis, the proteins associated with the actin filament organization are mainly up-regulated. As mentioned, OA is a potent phosphatase inhibitor and therefore able to increase the overall level of phosphorylation inside the cells. Therefore, phosphorylations may play a role in enzyme activity here. The analyzed phosphoproteins were also clustered according to the changes of the phosphorylations over time. Cluster 2 and 3 of the phosphoproteome include proteins associated with actin filament organization and cell–cell junction organization, respectively, but only the two proteins, myristoylated alanine-rich C-kinase substrate (MARCKS) and WASH complex subunit 2A (WASHC2A), were overlapping with the proteins associated with the cytoskeleton found regulated in the overall proteome analysis. To understand the depolymerization of F-actin observed upon OA exposure, many different factors and proteins have to be considered. First, actin itself is phosphorylated by P21-activated kinase 1 (PAK1), a kinase that is up-regulated in this study. This phosphorylation of actin can negatively influence the formation of the polymerized F-actin (Terman and Kashina 2013). Furthermore, F-actin is crosslinked by several different proteins that also depend on phosphorylation to exert their functions. One of those proteins is MARCKS, which is localized at the plasma membrane in its unphosphorylated state, where it is able to crosslink actin filaments. Upon activation by the protein kinase C (PKC), phosphorylated MARCKS then translocates into the cytoplasm, where it does no longer interact with actin (Fong et al. 2017). MARCKS is a major target of PKC (Blackshear 1993), and there is evidence that OA is able to prevent MARCKS dephosphorylation by inhibiting PP2A (Clarke et al. 1993). Our proteome dataset shows a decrease in overall MARCKS protein at the earlier timepoints 0.5 and 4 h, but an increase of overall MARCKS protein at 24 h. Furthermore, our phosphoproteome dataset also shows an increase of phosphorylated MARCKS depending on the time. This suggests an activation of MARCKS by PKC. However, the KSEA shows only a slight activation of the kinase activity of PKCα and β at 0.5 h and a decrease in PKCα and β phosphorylation at Thr641 for the other time points. The PKC isoforms all autophosphorylate themselves at this amino acid in their maturation process, and this phosphorylation is essential for PKC activity. Therefore, our results suggest an inhibition of PKC by OA. To be able to autophosphorylate, PKC has to be phosphorylated at Thr500 by a kinase called PDK1, which is also down-regulated in our DigiWest dataset, suggesting that the inactivation of PKC by OA might be due to an inactivation of PDK1 (Shirai and Saito 2002). However, the isoform PKCδ does not need the initial phosphorylation to autophosphorylate and be active. Furthermore, there is evidence that PKCδ is activated by reactive oxygen species (ROS). An increase in ROS upon OA exposure was previously reported in several studies, but seems to be dependent on the cell type (Túnez et al. 2003; Jayaraj et al. 2009; Valdiglesias et al. 2011). As the induction of ROS was detected as one of the top up-regulated pathways of the proteome dataset in the GO term analysis of this study, an activation of PKCδ by OA-induced ROS is possible. Therefore, because of this PKC isoform, MARCKS might be phosphorylated, even though most of the PKC isoforms are not activated.

Another important protein for F-actin crosslinking is zyxin (Crawford and Beckerle 1991). This protein was found up-regulated in cluster 7 of the shotgun dataset (Fig. 4), as well as in the previous 2D-study (Wuerger et al. 2023). Zyxin binds to vasodilator-stimulated phosphoprotein (VASP), a protein that is responsible for F-actin bundling and crosslinking (Bachmann et al. 1999). By binding to VASP, zyxin can act as a regulator of VASP-mediated actin regulation. There are two distinct binding sites of VASP to zyxin. While binding to one site alone cannot alter VASP activity, binding to both sites strongly reduces VASP-mediated actin regulation. This binding of VASP to zyxin can be regulated by phosphorylation of VASP (Grange et al. 2012). Furthermore, phosphorylation of VASP can, depending on the localization of the phosphorylation, alter VASP activity. While a phosphorylation at S157, mediated through protein kinase A (PKA), controls subcellular VASP distribution, phosphorylation at S239, mediated by protein kinase G (PKG) and to a lesser extent by PKA, and T278, mediated by AMP-activated protein kinase (AMPK), impair F-actin accumulation (Benz et al. 2009). There is evidence that exposure to OA leads to an activation of PKA by inhibiting PP2A (Sidhu and Omiecinski 1997). This activation in itself leads to an interference with actin polymerization (Ohta et al. 1987). Furthermore, OA is also able to activate AMPK (Samari et al. 2005), which we can confirm in HepaRG cells by our DigiWest dataset, where we see a clear increase in phosphorylated AMPK over. As PP2A inhibition also leads to AMPK activation (Dai et al. 2017), it can be assumed that AMPK activation by OA occurs through PP2A inhibition. This points to an inhibition of VASP activity by OA. Given the results of this study, the negative effect of OA on actin polymerization and crosslinking is mainly due to a dysregulation of phosphorylation patterns of the proteins involved in actin polymerization, which is a direct result of OA-induced PP2A inhibition.

The results obtained for several other proteins can also be directly traced back to PP2A inhibition. For example S6 kinase (RSK), a protein involved in translation, is directly dephosphorylated and thereby deactivated by PP2A (Wlodarchak and Xing 2016). This protein was found phosphorylated in our DigiWest dataset, while its primary target, ribosomal protein S6 (RPS6) (Ruvinsky and Meyuhas 2006), was found heavily phosphorylated in the same dataset. Furthermore, OA is a direct regulator of mitogen-activated protein kinase kinase (MEK) and extracellular-signal regulated kinase (ERK), two kinases of the MAPK signaling pathway (Millward et al. 1999). MEK1/2 and ERK2 were found phosphorylated at the earlier time points in our DigiWest dataset. Some proteins are connected to multiple functions within the cell. One example is AMPK, a protein that is activated by OA through PP2A inhibition (Samari et al. 2005; Dai et al. 2017). As already mentioned, AMPK plays a role in the destabilization of the cytoskeleton. However, its primary function is in energy metabolism. AMPK plays a key role in detecting adenosine monophosphate (AMP), which leads to AMPK activation and thereby to a regulation of several downstream factors to increase adenosine triphosphate (ATP) production and control ATP use. This leads to an inhibition of biosynthesis and an up-regulation of processes like lipolysis and gluconeogenesis (Herzig and Shaw 2018). As already mentioned, one of the top-down-regulated pathways of the GO term enrichment analysis of the transcriptome and proteome datasets is lipid metabolism. While some proteins that are part of lipid metabolism are indeed down-regulated, others show an up-regulation. Examples include acetyl-coenzyme A acyltransferase 1 (ACAA1) and peroxisomal acyl-coenzyme A oxidase 1 (ACOX1). However, those two are part of the peroxisomal β-oxidation, which is a process activated by AMPK (Herzig and Shaw 2018). We thereby hypothesize that the activation of AMPK by OA plays a key role in the observed alterations of lipid metabolism-related proteins.

## Conclusion

Overall, this study is the first of its kind to comprehensively decipher metabolic and signaling changes in human liver cells after exposure to OA, using a multi-omics approach. The results confirm the previous results regarding the down-regulation of xenobiotic metabolism by OA (Wuerger et al. 2022) and the negative effects on cytoskeleton polymerization (Dietrich et al. 2019). More importantly, however, they substantially expand our understanding of the underlying effects of these outcomes. In line with the phosphatase-inhibiting properties of OA, many observed effects include changes in the phosphorylation pattern of different proteins. This study now directly connects many of the previously observed effects by OA to its property to inhibit PP2A. It furthermore provides, for the first time, a multi-omics analysis of the effects of OA on human liver cells, revealing transcriptomic effects of OA, which had not been reported so far.

### Supplementary Information

Below is the link to the electronic supplementary material.

Supplementary file1 (PNG 468 KB)

Supplementary file2 (DOCX 1458 KB)

Supplementary file3 (XLSX 935 KB)

Supplementary file4 (XLSX 18 KB)

Supplementary file5 (XLSX 1444 KB)

## Data Availability

The raw RNA sequencing data are available from GEO under accession number GSE252563. The mass spectrometry proteomics data were deposited to the ProteomeXchange Consortium via the PRIDE partner repository with the identifier PXD048968. Further data are contained within the Supplementary Material or are available from the corresponding author upon reasonable request.
