# Supplementary figures and images for: A multi-omics approach to elucidate okadaic acid-induced changes in human HepaRG hepatocarcinoma cells
Source: Arch Toxicol. 2024 Jun 4;98(9):2919–35. doi: 10.1007/s00204-024-03796-1 (PMC11324782; doi:10.1007/s00204-024-03796-1)

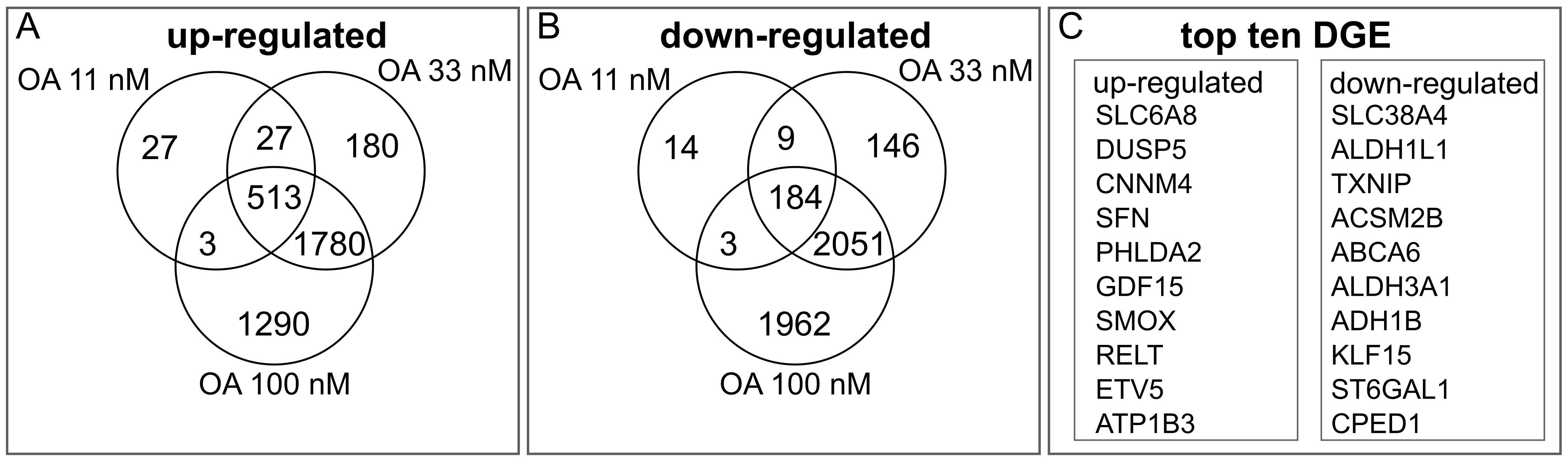

Supplement: Supplementary file 1 — Supplementary file1 (PNG 468 KB) [file 204_2024_3796_MOESM1_ESM.png]
