# Supplementary material for: A multi-omics approach to elucidate okadaic acid-induced changes in human HepaRG hepatocarcinoma cells
Source: Arch Toxicol. 2024 Jun 4;98(9):2919–35. doi: 10.1007/s00204-024-03796-1 (PMC11324782; doi:10.1007/s00204-024-03796-1)
Supplement: Supplementary file 2 — Supplementary file2 (DOCX 1458 KB) [file 204_2024_3796_MOESM2_ESM.docx]

**Supplemental data to:**

**A multi-omics approach to elucidate okadaic acid-induced changes in human HepaRG hepatocarcinoma cells**

In: Archives of Toxicology

Leonie T.D. Wuerger^a,$^, Heike Sprenger^a,$^, Ksenia Krasikova^a^, Markus Templin^b^, Uta M. Herfurth^a^, Holger Sieg^a^*, Albert Braeuning^a^

^a^ German Federal Institute for Risk Assessment, Department of Food Safety, Berlin, Germany

^b^ NMI Natural and Medical Sciences Institute, Reutlingen, Germany

^$^These authors contributed equally to the study and should both be considered first authors.

***Corresponding Author: Holger Sieg, holger.sieg@bfr.bund.de**

**E-Mail Addresses:**

Leonie T.D. Wuerger leonie.wuerger@bfr.bund.de

Heike Sprenger heike.sprenger@bfr.bund.de

Ksenia Krasikova ksek00@zedat.fu-berlin.de

Markus Templin markus.templin@nmi.de

Uta M. Herfurth uta.herfurth@bfr.bund.de

Holger Sieg holger.sieg@bfr.bund.de

Albert Braeuning albert.braeuning@bfr.bund.de

# Supplemental Tables

| ID | Sample Name | Condition | Total Raw Reads (million) | Total HQ Reads (million) | Mapped Reads (million) | Uniquely mapped Reads (million) | Percentage of Uniquely Mapped Reads (%) |
| --- | --- | --- | --- | --- | --- | --- | --- |
| 1 | LMK_1 | Negative control | 66 | 64.67 | 64.08 | 62.53 | 96.69 |
| 2 | LMK_2 | Negative control | 66 | 64.6 | 63.96 | 62.40 | 96.59 |
| 3 | LMK_3 | Negative control | 66 | 64.81 | 64.20 | 62.66 | 96.68 |
| 4 | OA_100_1 | OA 100 nM | 66 | 64.29 | 63.66 | 62.46 | 97.16 |
| 5 | OA_100_2 | OA 100 nM | 66 | 64.35 | 63.70 | 62.40 | 96.97 |
| 6 | OA_100_3 | OA 100 nM | 66 | 64.81 | 64.22 | 62.96 | 97.15 |
| 7 | OA_11_1 | OA 11 nM | 66 | 64.59 | 63.88 | 62.46 | 96.71 |
| 8 | OA_11_2 | OA 11 nM | 66 | 64.8 | 64.16 | 62.73 | 96.81 |
| 9 | OA_11_3 | OA 11 nM | 64.52 | 63.24 | 62.62 | 61.14 | 96.68 |
| 10 | OA_33_1 | OA 33 nM | 66 | 64.25 | 63.63 | 62.42 | 97.15 |
| 11 | OA_33_2 | OA 33 nM | 66 | 63.98 | 63.33 | 61.99 | 96.89 |
| 12 | OA_33_3 | OA 33 nM | 66 | 64.66 | 63.96 | 62.65 | 96.89 |

Supplemental Table 1. Summary of the RNA-Sequencing read data and 12 HepaRG cell samples treated by okadaic acid (OA). Raw reads were mapped to the human reference genome hg38 and uniquely mapped reads were used for further analyses.

*Supplemental Table 2. Overview of enriched GO terms for kmeans clusters of shotgun proteomics data set.*

- See file “Supplemental Table 2.xlsx”

| Concentration [nM] | Time [h] | Median of log_2_FC ratio |
| --- | --- | --- |
| 33 | 0.5 | 0.522 |
| 33 | 4 | 0.584 |
| 33 | 24 | 0.331 |
| 33 | **Overall** | **0.490** |
| 100 | 0.5 | 0.472 |
| 100 | 4 | 0.909 |
| 100 | 24 | 0.660 |
| 100 | **Overall** | **0.631** |

*Supplemental Table 3. Summary of log2FC ratio for phosphopeptides per time point and concentration after matching with shotgun proteomics data.*

*Supplemental Table 4. Results of Kinase-Substrate Enrichment Analysis (KSEA).*

- See file “Supplemental Table 4.xlsx”

*Supplemental Table 5. Results of Differential Gene Expression (DGE) analysis for significantly changed genes upon OA treatment.*

- See file “Supplemental Table 5.xlsx”

# Supplemental Figures


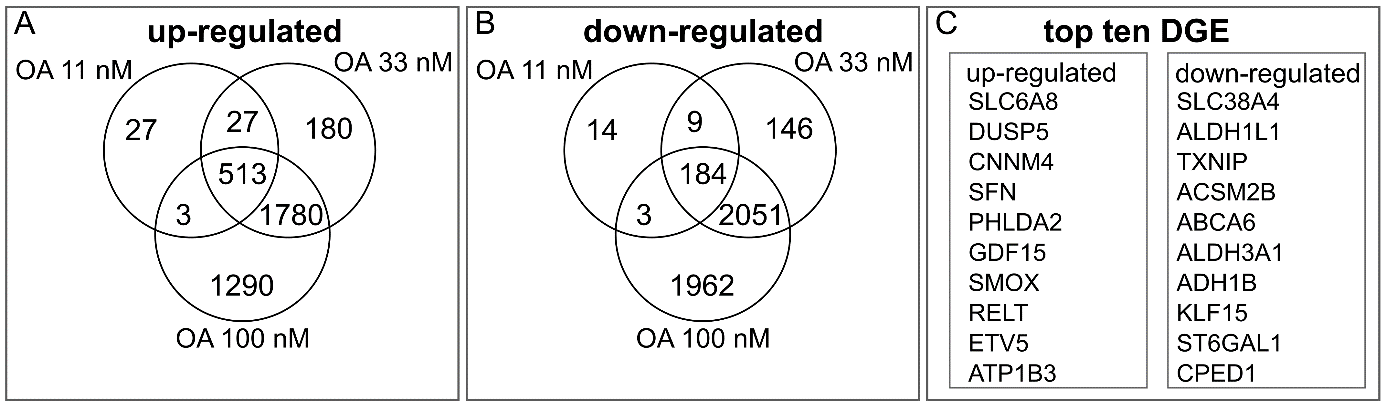


Supplemental Figure 1. Venn Diagram for significantly up-regulated (A) and down-regulated (B) genes upon OA treatment of 24 h measured by RNAseq. The top ten de-regulated genes of the overlap for OA 11 nM, 33 nM and 100 nM (ranked by log_2_FC values for OA 100 nM) are shown in subfigure C.


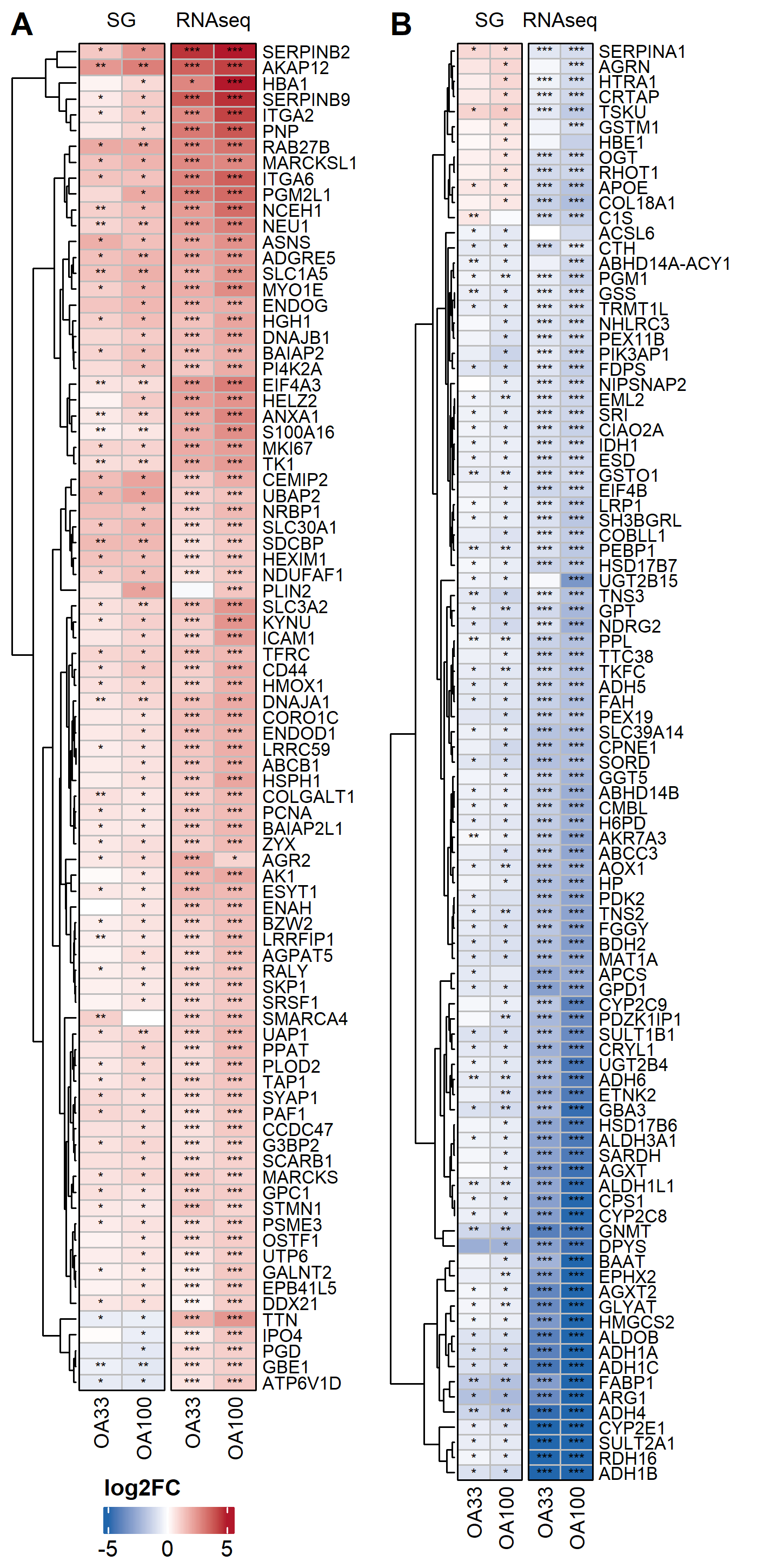


Supplemental Figure 2. Heatmap of log_2_FC-values for overlapping up-regulated (A) and down-regulated (B) genes/proteins measured by transcriptomics (RNAseq) and shotgun proteomics (SG). Only results for OA treatment (33, 100 nM) after 24 h are depicted. Statistical significance: ***p < 0.001, **p < 0.01, *p < 0.05.


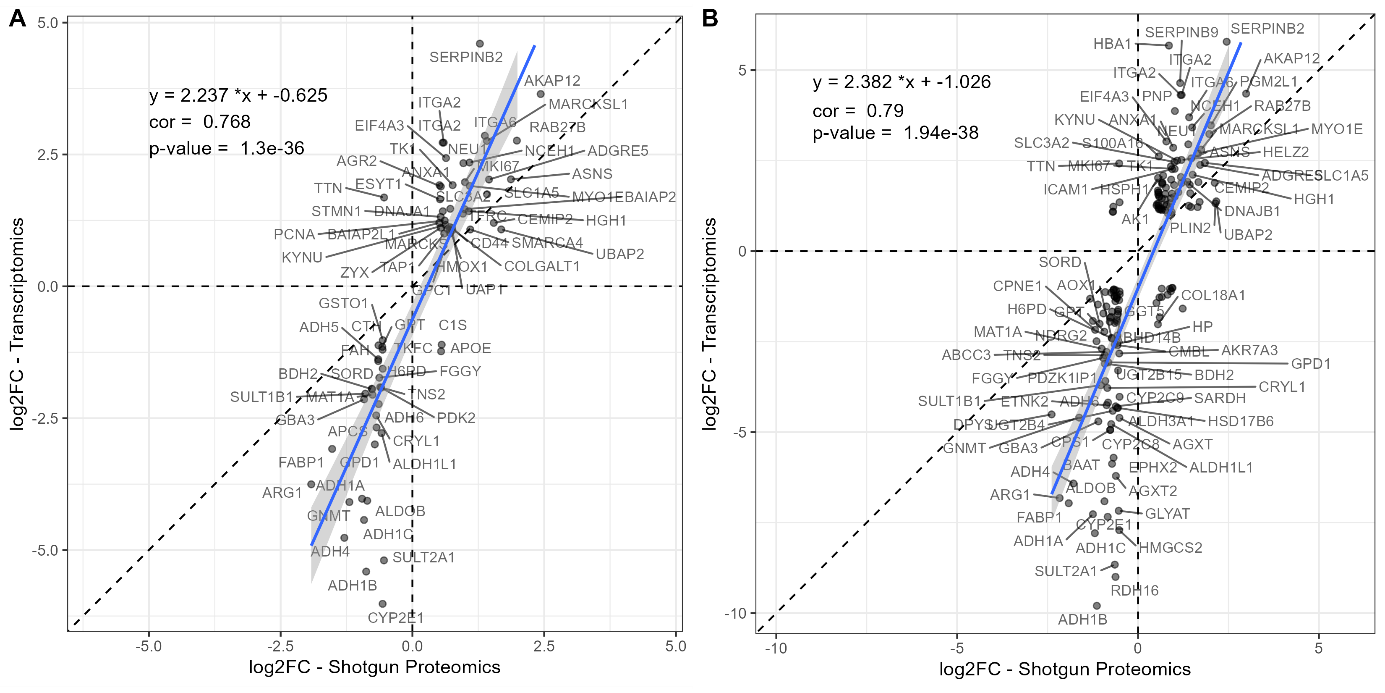


Supplemental Figure 3. Scatterplot of log2FC values for overlapping differentially regulated genes/proteins measured transcriptomics and shotgun proteomics. Results for treatment with OA 33 nM (A) and 100 nM (B) after 24 h are depicted. Correlation test was performed and summarized by Pearson correlation coefficient (cor) and p-value. Additionally, a linear model was fit with transcriptomics (y) ~ proteomics (x) and the equation derived.


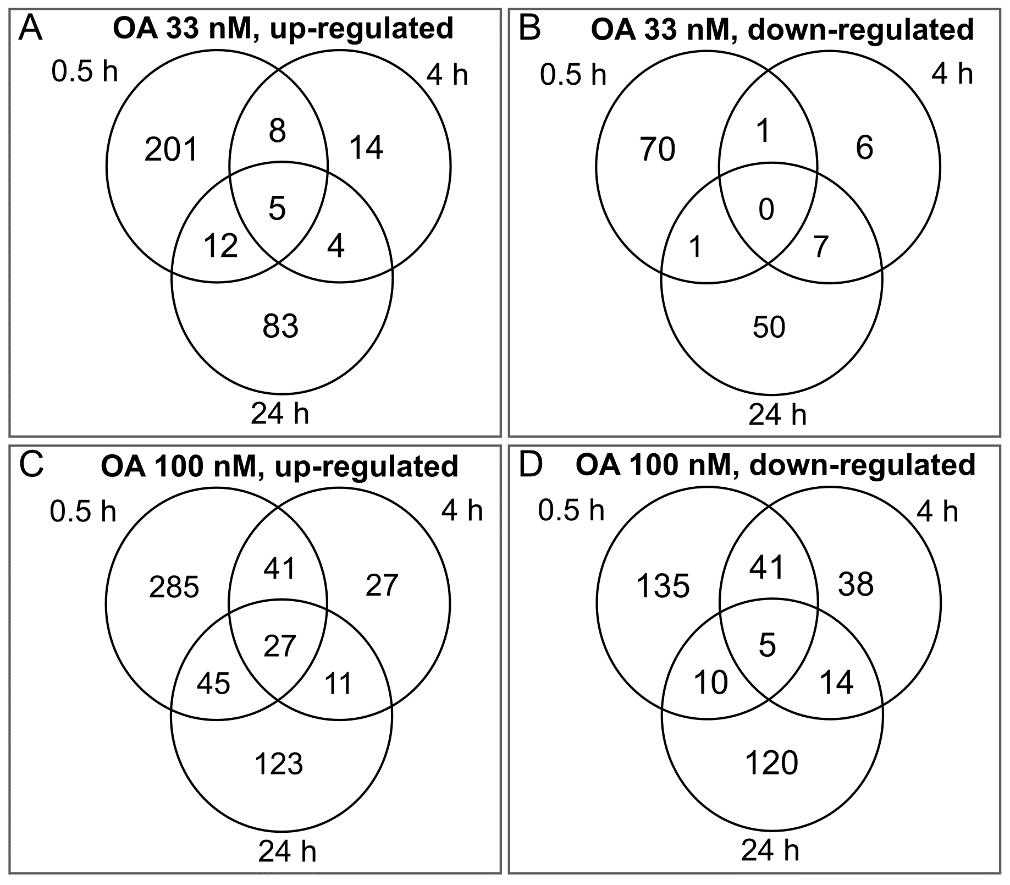


Supplemental Figure 4. Venn Diagram for significantly regulated proteins upon OA treatment (33 and 100 nM) for 0.5, 4 and 24 h measured by shotgun proteomics.


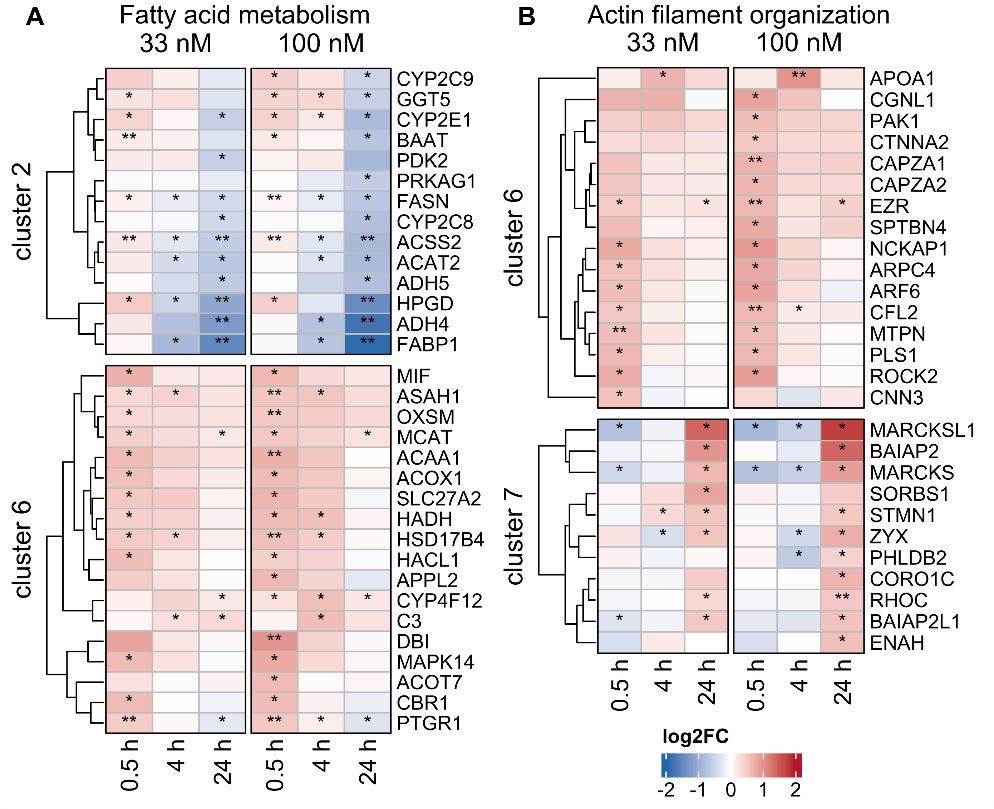


Supplemental Figure 5. Heatmap of log_2_FC-values of selected proteins from kmeans clustering for shotgun proteomics. Proteins were selected based on their annotation for the desired GO term “fatty acid metabolic process”, GO:0006631 (A) and “actin filament organization”, GO:0007015 (B). Statistical significance: ***p < 0.001, **p < 0.01, *p < 0.05.


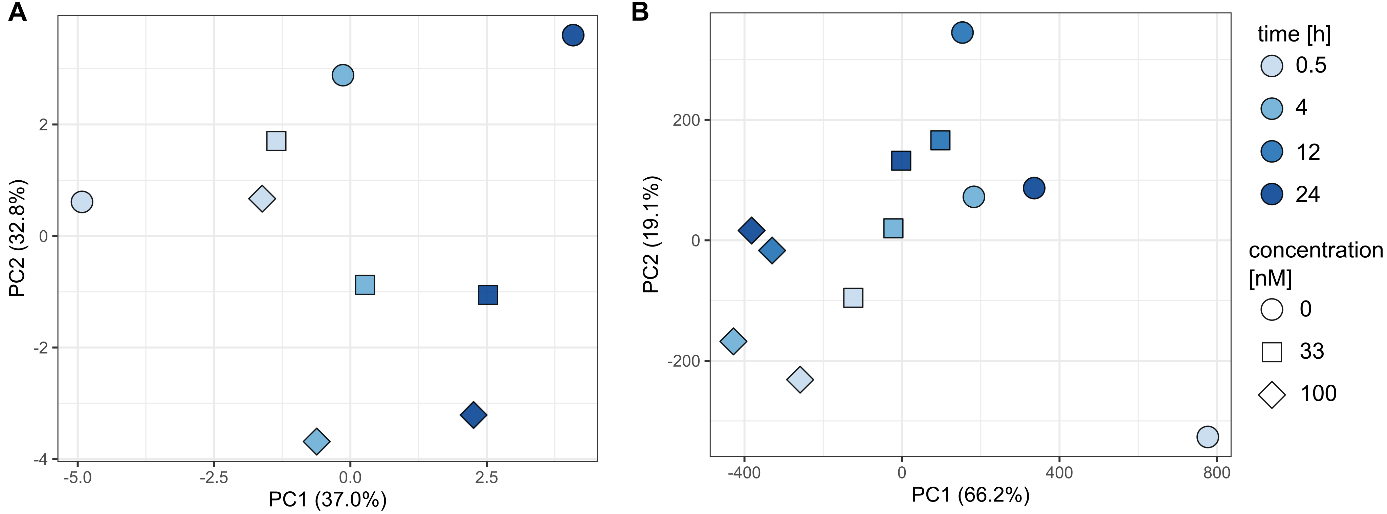


Supplemental Figure 6. PCA scores plot for phosphoproteome (A) and DigiWest (B) data set.


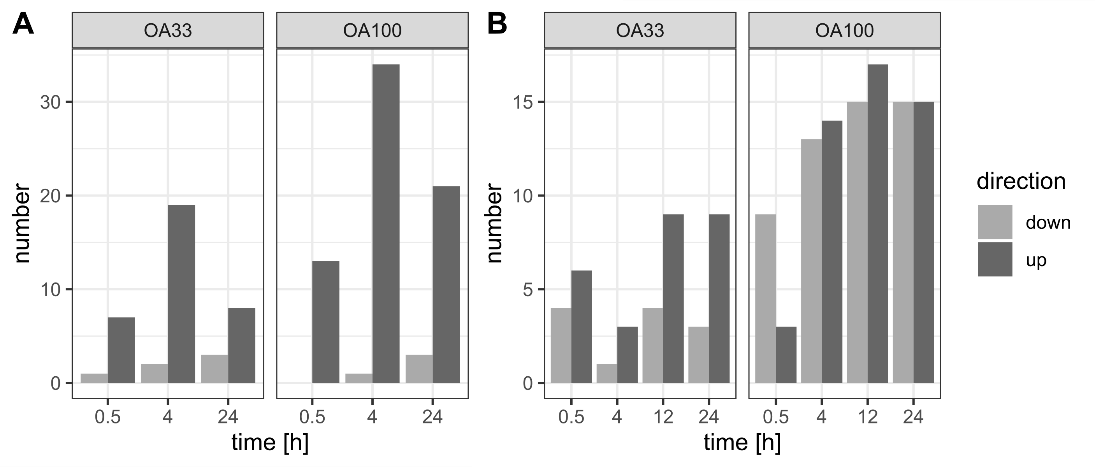


Supplemental Figure 7. Number of relevant changes in the phosphoproteome (A) and DigWest data set (B). The cut-off was |log2FC ratio| > 1 and |log2FC| > 1, respectively.


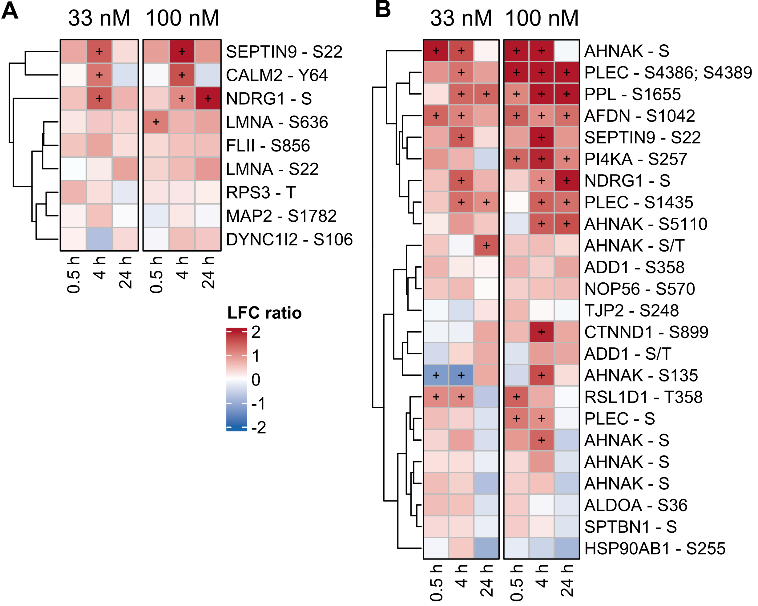


Supplemental Figure 8. Heatmaps of selected phosphopeptides related to microtubule (A) and cadherin binding (B). Relevant changes with |log2FC ratio| > 1 are indicated by “+” sign.


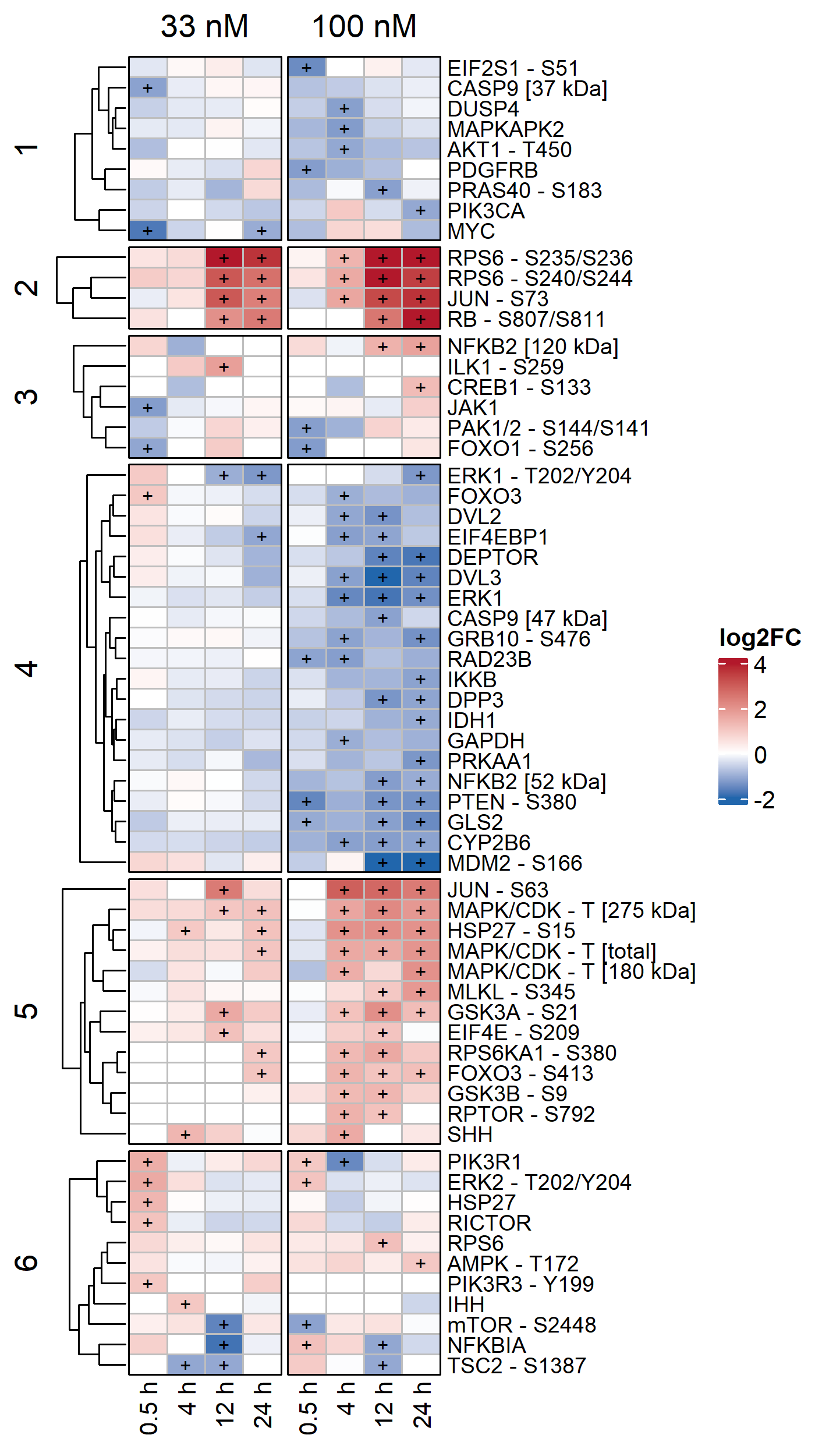


Supplemental Figure 9. Heatmap for subset of "relevant" analytes measured by DigiWest and split according to their kmeans cluster grouping. Relevant changes with |log2FC ratio| > 1 are indicated by “+” sign.


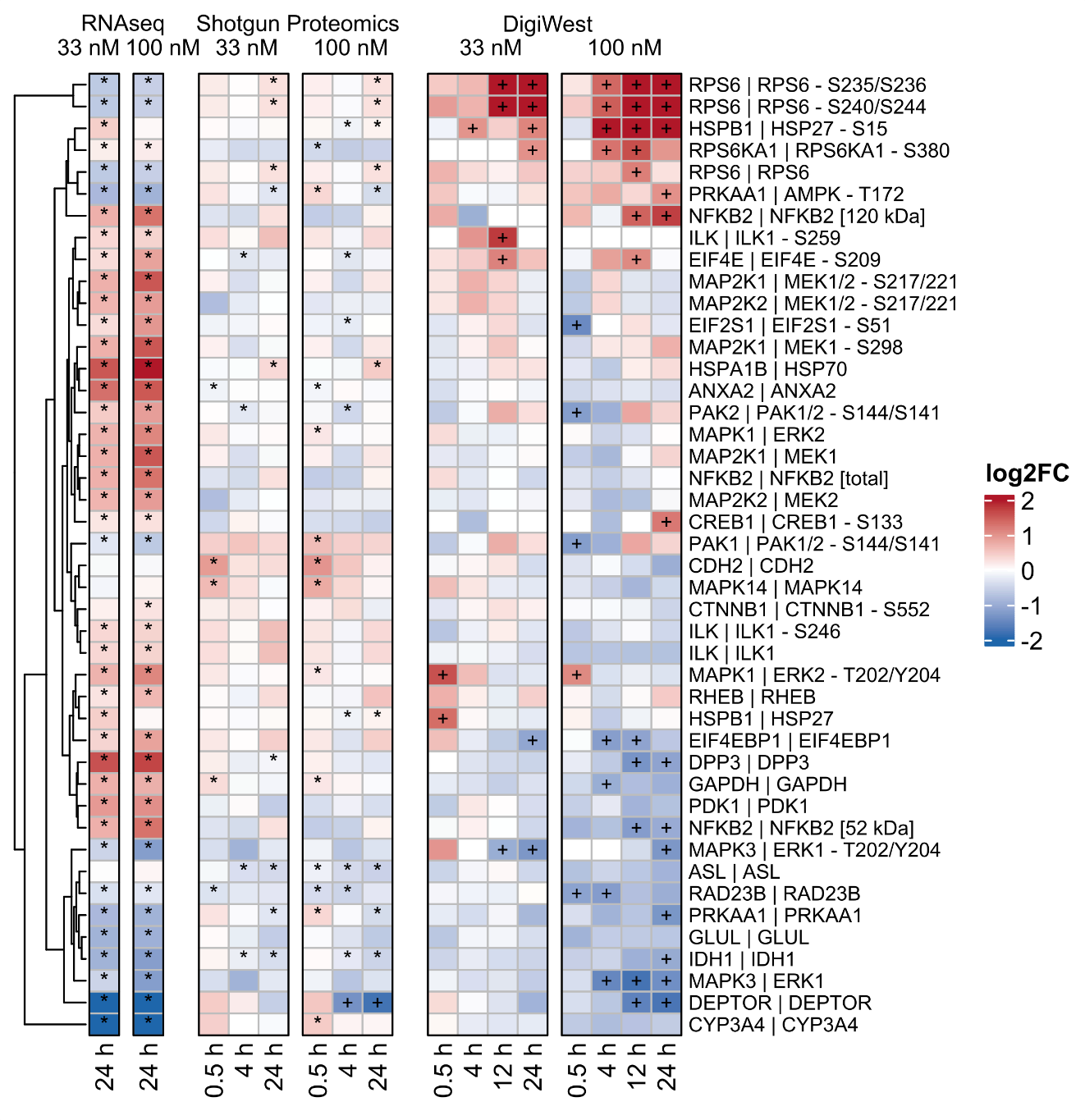


Supplemental Figure 10. Combined heatmap of overlapping genes/proteins/analytes for transcriptomics (RNAseq), shotgun proteomics and DigiWest analyses. Significant changes for RNAseq and Shotgun Proteomics are indicated by * sign, while relevant changes for DigiWest are indicated by “+” sign (if |log2FC ratio| > 1).
